# Supplementary material for: Mutational Processes Molding the Genomes of 21 Breast Cancers
Source: Cell. 2012 May 25;149(5-10):979–93. doi: 10.1016/j.cell.2012.04.024 (PMC3414841; doi:10.1016/j.cell.2012.04.024)
Supplement: Document S2. Article plus Supplemental Information [file mmc3.pdf]

Cell

Mutational Processes Molding the Genomes of 21 Breast Cancers

Serena Nik-Zainal,[1] Ludmil B. Alexandrov,[1] David C. Wedge,[1] Peter Van Loo,[1,2,3] Christopher D. Greenman,[1,4,5] Keiran Raine,[1] David Jones,[1] Jonathan Hinton,[1] John Marshall,[1] Lucy A. Stebbings,[1] Andrew Menzies,[1] Sancha Martin,[1] Kenric Leung,[1] Lina Chen,[1] Catherine Leroy,[1] Manasa Ramakrishna,[1] Richard Rance,[1] King Wai Lau,[1] Laura J. Mudie,[1] Ignacio Varela,[1] David J. McBride,[1] Graham R. Bignell,[1] Susanna L. Cooke,[1] Adam Shlien,[1] John Gamble,[1] Ian Whitmore,[1] Mark Maddison,[1] Patrick S. Tarpey,[1] Helen R. Davies,[1] Elli Papaemmanuil,[1] Philip J. Stephens,[1] Stuart McLaren,[1] Adam P. Butler,[1] Jon W. Teague,[1] Göran Jönsson,[13] Judy E. Garber,[7] Daniel Silver,[7] Penelope Miron,[7] Aquila Fatima,[7] Sandrine Boyault,[8] Anita Langerød,[9] Andrew Tutt,[10] John W.M. Martens,[11] Samuel A.J.R. Aparicio,[6,12] Åke Borg,[13] Anne Vincent Salomon,[14] Gilles Thomas,[8] Anne-Lise Børresen-Dale,[9,15] Andrea L. Richardson,[7,16] Michael S. Neuberger,[17] P. Andrew Futreal,[1] Peter J. Campbell,[1,18,19] Michael R. Stratton[1,*] and the Breast Cancer Working Group of the International Cancer Genome Consortium

[1]Cancer Genome Project, Wellcome Trust Sanger Institute, Hinxton CB10 1SA, UK
[2]Center for the Biology of Disease, VIB, Herestraat 49 Box 602, B-3000 Leuven, Belgium
[3]Department of Human Genetics, KU Leuven, Herestraat 49 Box 602, B-3000 Leuven, Belgium
[4]Department of Computing, University of East Anglia, Norwich NR4 7TJ, UK
[5]The Genome Analysis Centre, Norwich Research Park, Norwich NR4 7UH, UK
[6]Department of Pathology and Laboratory Medicine, University of British Columbia, Vancouver, BC V6T 2B5, Canada
[7]Dana-Farber Cancer Institute, 450 Brookline Avenue, Boston, MA 02215, USA
[8]Faculté de Médecine, INCa-Synergie, Centre Leon Berard, 28 rue Laennec, Lyon Cedex 08, France
[9]Department of Genetics, Institute for Cancer Research, Oslo University Hospital, The Norwegian Radium Hospital, O310 Oslo, Norway
[10]Breakthrough Breast Cancer Research Unit, Kings College, London SE1 9RT, UK
[11]Department of Medical Oncology, Erasmus Medical Center, Postbus 2040, 3000 CA Rotterdam, The Netherlands
[12]Molecular Oncology, British Columbia Cancer Research Centre, Vancouver, BC V5Z 1L3, Canada
[13]Department of Oncology, Lund University, BMC C13, SE-221 84 Lund, Sweden
[14]Institut Curie, Departement of Pathology and INSERM U830, 75248 Paris Cedex 05, France
[15]K.G. Jebsen Center for Breast Cancer Research, Institute for Clinical Medicine, Faculty of Medicine, University of Oslo, Oslo 0310, Norway
[16]Brigham and Women's Hospital, Harvard Medical School, 75 Francis St, Boston, MA 02115, USA
[17]MRC Laboratory of Molecular Biology, Cambridge CB2 0QH, UK
[18]Department of Haematology, Addenbrooke's Hospital, Cambridge CB2 0QQ, UK
[19]Department of Haematology, University of Cambridge, Cambridge CB2 2XY, UK
*Correspondence: mrs@sanger.ac.uk
DOI 10.1016/j.cell.2012.04.024

SUMMARY

All cancers carry somatic mutations. The patterns of mutation in cancer genomes reflect the DNA damage and repair processes to which cancer cells and their precursors have been exposed. To explore these mechanisms further, we generated catalogs of somatic mutation from 21 breast cancers and applied mathematical methods to extract mutational signatures of the underlying processes. Multiple distinct single- and double-nucleotide substitution signatures were discernible. Cancers with *BRCA1* or *BRCA2* mutations exhibited a characteristic combination of substitution mutation signatures and a distinctive profile of deletions. Complex relationships between somatic mutation prevalence and transcription were detected. A remarkable phenomenon of localized hypermutation, termed "kataegis," was observed. Regions of kataegis differed between cancers but usually colocalized with somatic rearrangements. Base substitutions in these regions were almost exclusively of cytosine at TpC dinucleotides. The mechanisms underlying most of these mutational signatures are unknown. However, a role for the *APOBEC* family of cytidine deaminases is proposed.

INTRODUCTION

Cancers carry somatic mutations. A small proportion are "drivers" that confer clonal advantage, are causally implicated in oncogenesis, and have been positively selected during the evolution of the cancer (Stratton, 2011; Stratton et al., 2009). Driver mutations occur in the subset of genes known as cancer genes. Through systematic sequencing of cancer genomes, considerable advances have recently been made in the identification of cancer genes, providing insights into mechanisms of neoplastic transformation and targets for therapeutic intervention (Stratton, 2011; Stratton et al., 2009). We have relatively limited understanding, however, of the DNA damage and repair processes that have been operative during the lifetime of the

patient and that are responsible for the somatic mutations that underlie the development of all cancers in the first place.

Historically, analysis of mutation patterns to investigate underlying DNA damage and repair processes in human cancers has predominantly been restricted to reporter cancer genes, notably *TP53*. These studies have revealed that mutation patterns can be related to carcinogen exposures and DNA repair processes. For example, G>T/C>A transversions predominate in smoking-associated lung cancer, a pattern compatible with DNA damage induced by tobacco carcinogens such as benzo[a]pyrene diolepoxide (Pfeifer et al., 2002). These mutations are enriched at CpG dinucleotides and exhibit a transcriptional strand bias reflecting past activity of transcription-coupled nucleotide excision repair (TCR) on bulky adducts of guanine caused by tobacco carcinogens (Hainaut and Pfeifer, 2001). Similarly, in UV-light-associated skin cancers, C>T and CC>TT transitions are common. These occur at dipyrimidines, reflecting the formation of pyrimidine dimers following exposure of DNA to UV light (Pfeifer et al., 2005) and also show transcriptional strand bias due to the action of TCR on pyrimidine dimers. Further examples of exogenous exposures leading to distinctive mutational patterns include G>T transversions in aflatoxin B1-associated hepatocellular carcinomas (Macé et al., 1997) and A>T transversions in urothelial tumors from patients exposed to aristolochic acid (Nedelko et al., 2009).

Although these studies have been highly informative, they have limitations. Because they depend upon driver mutations, effects of selection have been superimposed upon mutational patterns generated by DNA damage and repair processes. Moreover, only a single mutation from each cancer sample is usually incorporated into each data set. Thus, they have been well placed to report strong exposures and dominant repair processes operative across most cases of a particular tumor type. However, where there is heterogeneity of mutational process in a cancer class, a composite of different processes will be reported.

The completion of the human genome sequence and the advent of second-generation sequencing technology have overcome historic limitations of scale, permitting sequencing of whole cancer genomes (Berger et al., 2011; Chapman et al., 2011; Ding et al., 2010; Lee et al., 2010; Ley et al., 2008; Mardis et al., 2009; Shah et al., 2009; Tao et al., 2011), and generation of comprehensive catalogs of somatic mutation (Pleasance et al., 2010a; Pleasance et al., 2010b). Most somatic mutations in cancers are thought to be “passenger” events that do not contribute to cancer development. These bystanders bear the imprints of the DNA damage and repair processes operative during the development of the cancer, unmodified by selection. The several hundreds to tens of thousands of somatic mutations in each cancer, therefore, potentially allow much greater resolution of mutational patterns and insights into underlying mutational processes.

Recent analyses of comprehensive mutational catalogs from a malignant melanoma and a lung cancer illustrate the power of this approach (Pleasance et al., 2010a; Pleasance et al., 2010b). They revealed the characteristic mutational patterns of UV light and tobacco carcinogens respectively (see above) and provided strong evidence for the past activity of TCR. In addition, G>T mutations in the lung cancer showed a preference for CpG dinucleotides outside CpG islands, suggesting a role for methylated cytosine because CpG islands are usually unmethylated. Conversely, G>C mutations at CpG dinucleotides were more prevalent within CpG islands suggesting that the mutagen(s) underlying these mutations preferentially acted on unmethylated DNA (Pleasance et al., 2010b). In the melanoma, at least one additional mutational process characterized by G>T changes and independent of UV light exposure was shown to have been operative. In both cancers, mutations were more common in poorly expressed than in highly expressed genes, both on the transcribed and untranscribed strands. Indeed, mutations were also found to be more prevalent at the 3′ ends of genes than at the 5′ ends (Pleasance et al., 2010a). The mechanisms underlying these expression-related phenomena are unknown.

Compared to melanoma and lung cancer, the mutational processes underlying other cancer types are poorly understood. Therefore, in this study, we document essentially the full repertoire of somatic mutations of 21 breast cancers to investigate the mutational mechanisms shaping these cancer genomes.

RESULTS

Sequencing of Breast Cancers

We sequenced the complete genomes of 21 primary breast cancers and matched normal DNAs from the same individuals. Cancers were typed for estrogen receptors (ER), progesterone receptors (PR), and HER2, and included nine cases with germline mutations in the breast cancer predisposition genes *BRCA1* (five) and *BRCA2* (four) (Table S1A, available online).

Cancer and normal DNAs were sequenced to > 30-fold coverage and analyzed to identify somatic base substitutions, insertions, and deletions (indels); rearrangements; and copy number changes. PD4120a was sequenced to ~188-fold depth to investigate temporal and clonal evolution (Nik-Zainal et al., 2012). Using orthogonal sequencing technologies we estimated the specificity of substitution-calling to be ~92.1% (Table S1A). All substitutions were therefore included in the analyses. For indels and rearrangements only confirmed variants were included (Table S1B). From 17 of the 21 cases mRNA expression data were also obtained.

The Catalogue of Somatic Mutations from 21 Breast Cancer Genomes

A total of 183,916 somatically acquired base substitutions were identified (see Table S1B for hyperlinks). In protein coding regions, there were 1,372 missense, 117 nonsense, 2 stop-lost, 37 essential splice-site, and 521 silent mutations. Of the 2,869 indels identified, 2,233 were deletions, 544 insertions and 92 complex. There were 21 coding indels, of which 15 were predicted to result in a translational frameshift and six were in-frame. In addition, 1,192 structural variants (rearrangements), 16 homozygous deletions, and 14 regions of increased copy number (amplifications) were identified (Table S1C).

Likely driver substitutions and indels in cancer genes were found in *TP53*, *GATA3*, *PIK3CA*, *MAP2K4*, *SMAD4*, *MLL2*, *MLL3*, and *NCOR1* (Table S1C). Amplification was

observed over cancer genes previously implicated in breast cancer development including *ERBB2, CCND1*, *MYC*, *MDM2*, *ZNF217*, and *ZNF703* and a homozygous deletion involving *MAP2K4* was identified. All tumors derived from *BRCA1* or *BRCA2* germline mutation carriers showed loss of wild-type haplotypes at 17q21 or 13q12, respectively, as expected of recessive cancer genes (Table S1B).

Extracting Mutation Signatures from Catalogues of Somatic Mutation

The set of somatic mutations in a cancer genome is the aggregate outcome of one or more mutational processes. Each process leaves a mutation signature on the cancer genome defined by the mechanisms of DNA damage and repair that constitute it. The final catalog of mutations is determined by the strength and duration of exposure to each mutational process. We set out to extract the mutation signatures characterizing the mutational processes operative in the 21 breast cancers studied.

There was substantial variation between the cancers in the numbers and relative contributions of each of the six classes of base substitution (C>A, C>G, C>T, T>A, T>C, and T>G) (Figure 1A). To provide greater depth of insight into the operative mutational processes, we incorporated the sequence context in which mutations occurred, by considering the bases immediately 5′ and 3′ to each mutated base. Because there are six classes of base substitution and 16 possible sequence contexts for each mutated base there are 96 possible mutated trinucleotides. We have represented the fraction of mutations at each of the 96 mutated trinucleotides as a heat map for each cancer and normalized it according to the prevalence of each trinucleotide in the genome. The display therefore highlights mutational signatures generated by processes that favor particular classes of mutation and/or particular sequence contexts in which mutations occur (Figure 1C).

Visual inspection of the 21 heatmaps provided evidence for multiple independent mutational signatures and indicated that, in most cancers, more than one process had been operative. For example, overrepresentation compared to chance of C>T substitutions at XpCpG triplets (C is the mutated base and X is any base) was observed in all cancers, albeit to different extents. The elevated C>T mutation rate at XpCpG trinucleotides is a well-recognized mutational mechanism probably due to deamination to thymine of methylated cytosines, which are usually at XpCpGs (Waters and Swann, 2000). The role of methylated cytosine is supported in our data by the higher frequency of C>T transitions at XpCpG triplets outside CpG islands (where most XpCpGs are methylated) than inside (where most are unmethylated) (OR 9.95; 95% CI 7.17–13.8; $p < 0.0001$). Subtler features of this mutational signature were also apparent, notably the influence of the base 5′ to the mutated cytosine on the C>T mutation rate (for example, see PD3905a). Although the normalized heatmap representation emphasizes the ubiquitous elevation of the C>T mutation rate at XpCpG trinucleotides, the absolute number of these mutations is relatively modest because of the general depletion of XpCpGs from the human genome due to the activity of the same, or a similar, mutational process in the germline over evolutionary time.

There was also an overrepresentation of C>T, C>G and C>A mutations at TpCpX trinucleotides in many breast cancers and very pronounced in some (notably, PD4199a and P4120a). In addition to the high proportion of T immediately 5′ to the mutated cytosine, the base immediately 3′ to the mutated C also appears to influence this mutational signature with greater overrepresentation of TpCpA, TpCpT and TpCpG than of TpCpC. We have previously reported this signature in breast and other cancers (Greenman et al., 2007; Stephens et al., 2005, 2012).

Application of Mathematical Approaches to Extract Mutation Signatures

Although major features of some mutational signatures can be discerned by visual inspection, a formal mathematical approach is required to extract subtler elements and weaker signatures and to assess the contribution of each mutational process to the mutation set in each cancer. We employed a nonnegative matrix factorization (NMF) and model selection approach (Berry et al., 2007) to extract mutational signatures from the 21 cases. NMF extracts interpretable features from complex multidimensional data (Berry et al., 2007; Lee and Seung, 1999). For example, application to images of faces yields familiar components such as eyes, nose, and mouth (Lee and Seung, 1999). Our desire to extract biologically meaningful mutational signatures, as well as the intrinsic nonnegativity of the mutation spectrum data, renders NMF an appropriate choice for factorizing the data from the 21 cases.

Evaluation of NMF decompositions (Berry et al., 2007) (Extended Experimental Procedures and Figures S1A–S1C) suggested that a best estimate of five biologically distinct mutational signatures were present in the 21 cancers (named A–E, Figure 2A). Each signature was characterized by a different profile of the 96 potential trinucleotide mutations and contributed to a different extent to each of the 21 cancers. Different combinations of the five signatures account for the variation in the 21 mutational catalogs (Figure 1D).

Signature A was characterized by C>T mutations at XpCpG trinucleotides but included other mutation classes making smaller contributions (Figure 2A). Signature B was composed predominantly of C>T, C>G and C>A mutations at TpCpX trinucleotides. The dominant features of these two mutational signatures were previously noted by scrutiny of the heatmaps (see above). However, for each process NMF provided greater insight into the relative contributions of all mutation classes.

NMF provided evidence for three additional mutational processes. Signature C and Signature D both exhibited a relatively even distribution of mutations across the 96 trinucleotides. However, there were subtle differences. Signature C was moderately enriched for C>T, C>G and, to a lesser extent, C>A mutations at XpCpG trinucleotides, whereas Signature D was not (Figures 2A and S1D). In hindsight, an enrichment of C>G and C>A mutations at XpCpG trinucleotides can be discerned in some cancers in the heatmap (Figure 1C). Moreover, the strength of this enrichment does not appear to be well correlated with enrichment of C>T mutations at XpCpG trinucleotides, suggesting that they are due to different processes, providing the rationale for NMF to separate Signature C from Signature A

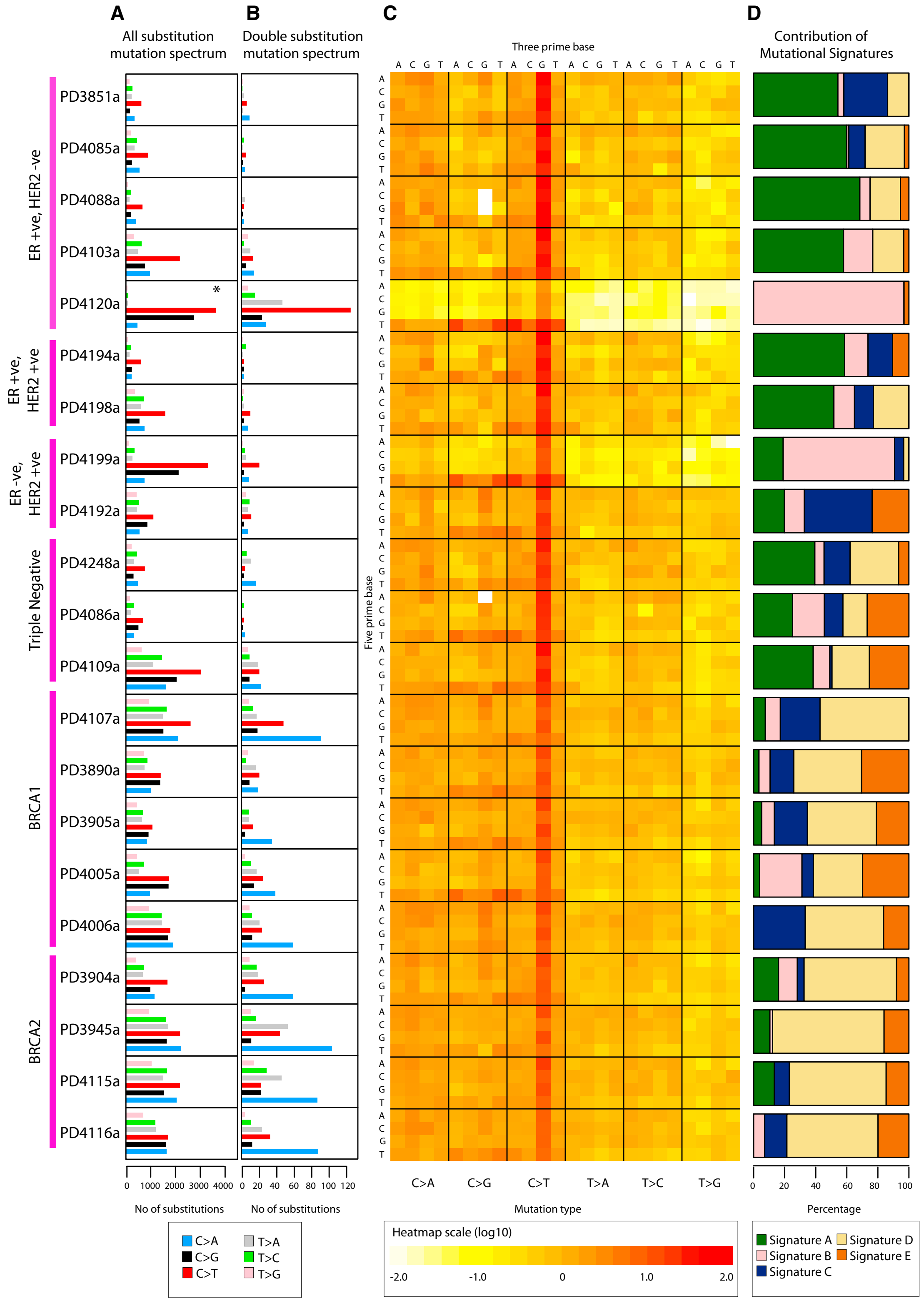

Figure 1. Somatic Mutation Profiles of 21 Breast Cancers, Related to Table S1

Breast cancers grouped according to subtype on the far left.

(A) Base substitution mutation spectra. *Ultra-deep sequenced PD4120a has an alternative scale on the x axis (0 to 45,000).

(B) Mutation spectra of double substitutions from all 21 samples.

(compare, for example, PD4006a and PD3945a in Figure 1C). In Signature E the dominant feature was C>G mutations at TpCpX trinucleotides. Signature E is therefore similar to Signature B, but lacks the C>T mutations at TpCpX trinucleotides characteristic of Signature B.

NMF will extract mutational patterns due to sequencing artifacts. We noted a signature characterized by T>G mutations at GpTpX trinucleotides. This was not, however, reproduced in somatic mutations verified by using another sequencing technology and transpired to be due to aberrant sequence phasing at Ts following runs of Gs in the genome.

NMF can estimate the contribution of each mutational signature to the mutational catalog of each breast cancer. The results indicate that multiple mutational processes contribute to most cancers, although in some cases one process has been dominant (Figure 1D). No correlation was found between the presence of a particular somatically mutated gene and any of these processes. We then performed unsupervised hierarchical clustering by using the relative contributions of each of the five signatures to each of the 21 mutational catalogs as features. Interestingly, all nine breast cancers with *BRCA1* or *BRCA2* mutations clustered together (Figure 2B). This was predominantly due to a relatively substantial contribution by Signature D and a relative deficiency of Signature A in these cancers.

Mutational Processes Generating Double-Nucleotide Substitutions

By performing Monte Carlo simulations we showed that the number of double-nucleotide substitutions (for example, CpC>ApT) in each of the 21 cancer genomes was 75–11,000 fold higher than expected if single-nucleotide substitutions had been randomly distributed ($p < 0.001$) (Table S1D). Thus, a mutational process generating double-nucleotide substitutions seems to be ubiquitous. Overall, the patterns of double-nucleotide substitutions reflected those of single-nucleotide substitutions in each cancer. However, in most cases there was substantial enrichment of C>A substitutions as components of double-nucleotide substitutions (Figure 1B) with the consequent emergence of CpC>ApA as the most common class of double-nucleotide substitution (Table S1E). Double-nucleotide substitutions were distributed throughout the cancer genomes without obvious evidence of further clustering.

Regional Hypermutation, *Kataegis*, Is Common in Breast Cancer

We investigated the possibility of regional clustering of substitution mutations by constructing ‘‘rainfall plots’’ in which the intermutation distance, the distance between each somatic substitution, and the substitution immediately before it has been plotted for each mutation (Figures 3A–3C and S2). Mutation clustering was commonly observed in the 21 breast cancers. The mutational patterns within these clusters are outlined below by using two cases as examples.

The largest regional cluster of mutations was found in a breast cancer with a germline mutation in *BRCA1*, PD4107a, which showed a markedly elevated mutation prevalence over a 14 Mb region on chromosome 6 (Figure 3A). The mutations within this cluster exhibited several remarkable features. First, there was evidence of further clustering within the 14 Mb region (the ‘‘macrocluster’’), with heavily mutated stretches of genome of a few hundred base pairs (‘‘microclusters’’) sometimes separated by tens of kilobases without mutations (Figure 4A). Second, substitutions within the region were characterized by a distinctive mutational spectrum and sequence context (Figure 3D). Most were C>T transitions at TpCpX trinucleotides. Third, examination of individual sequence reads, which derive from individual DNA molecules, showed that most mutations within microclusters occurred in *cis* with respect to each other, i.e., were on the same parental chromosome (Figure 4A). Fourth, mutations were generally of the same type for long genomic distances and then could switch to a different class. For example, in PD4107a mutations were almost exclusively C>T (on the plus chromosomal strand) for several megabases and then switched to G>A (Figure 4B). This suggests that groups of mutations may be generated on just one of the two DNA strands, perhaps simultaneously or in a processive manner over a short time span. Fifth, the cluster of mutations on chromosome 6 colocalizes with a cluster of somatic genomic rearrangements (Figure 4A). Within the hypermutated 14 Mb region, there were 17 genomic rearrangements but only seven in the remaining 157 Mb of chromosome 6. Most of these rearrangements were between different locations within the chromosome 6 14 Mb region. Despite the clear positional correlation between rearrangements and hypermutation, at higher resolution mutation microclusters were usually separated from the nearest rearrangement by many kilobases. Finally, in PD4107a a much smaller mutation cluster with similar mutational characteristics and associated with genomic rearrangements was observed on chromosome 12.

An ER-positive breast cancer, PD4103a, also exhibited localized hypermutation, but the pattern of mutation clustering differed slightly from that in PD4107a (Figure 3B). There were multiple mutation clusters involving chromosomes 3, 4, 8, 10, 11, 12, 20, and 21 each of which spanned shorter distances than the major cluster in PD4107a. The clustered substitutions in PD4103a included C>T transitions at TpCpX dinucleotides, similar to PD4107a, but in addition a greater proportion of C>G mutations at TpCpX trinucleotides. In other respects, notably the mutations being in *cis* and showing a processive pattern, there were many similarities (Figures 5A–5B). Mutation clusters in PD4103a were also closely associated with somatic genomic rearrangements that were all linked together by a web of interchromosomal rearrangements (Figure 5C).

We have termed the presence of regional mutation clusters *kataegis* (from Greek for shower or thunderstorm). Rainfall plots revealed varying extents of kataegis in 13/21 breast cancers (PD4199a, PD4192a, PD4198a, PD4248a, PD4109a, PD4116a,

(C) Genomic heatmap constructed from counts of each mutation-type at each mutation context corrected for the frequency of each trinucleotide in the reference genome. Log-transformed values of these ratios have been plotted in the heatmap. The 5′ base to each mutated base is shown on the vertical axis and 3′ base on the horizontal axis. Heatmap scale at the bottom.

(D) Proportion of the total substitutions contributed by each of the five mutational signatures, as identified by NMF analysis, for all 21 cancer genomes.

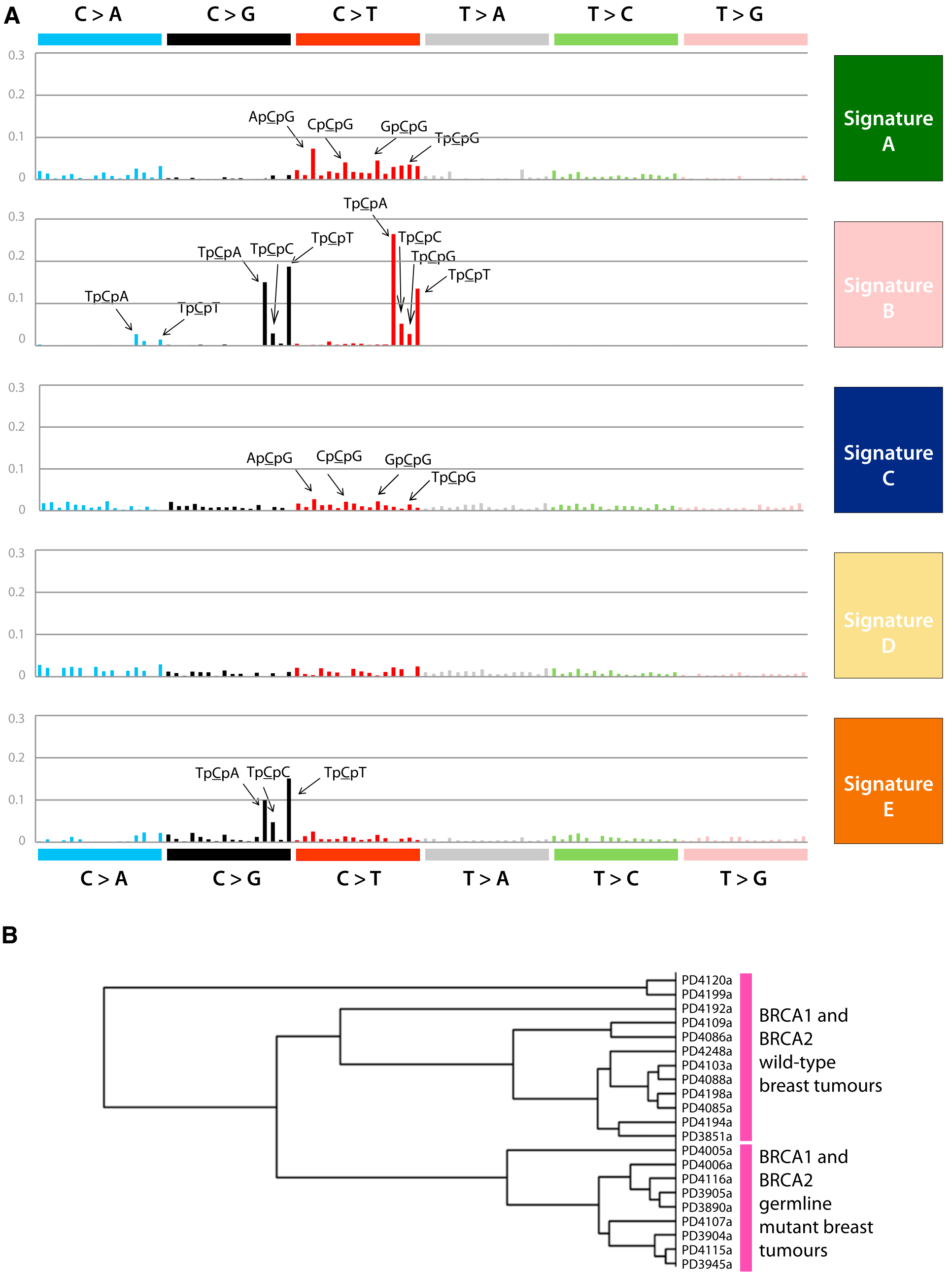

Figure 2. Five Mutational Signatures Extracted by NMF in 21 Breast Cancers, Related to Figure S1

(A) Fraction of contribution of each mutation-type at each context for the five mutational signatures identified by NMF analysis. The major components contributing to each signature are highlighted with arrows.

(B) Cluster dendrogram generated by unsupervised hierarchical clustering based on contributions of the five mutational signatures identified by NMF to the 21 breast cancer genomes.

PD3904a, PD3945a, PD4005a, PD4006a, PD4103a, PD4120a, and PD4107a, see Figure S2), encompassing all subclasses of the disease. In each case, the features were similar to those outlined for PD4107a and PD4103a. In some instances, kataegis was associated with rearrangements that had features of chromothripsis (Stephens et al., 2011) (Figure 4D), but it also colocalized with other rearrangement architectures. Previously published mutation catalogs from a malignant melanoma and small cell lung cancer did not show kataegis (Pleasance et al., 2010a, 2010b) (data not shown).

The pattern of C>T and C>G mutations at TpCpX trinucleotides in kataegis is similar to that of mutational Signature B, and to a lesser extent, Signature E (Figure 2A). Yet, in many cancers with kataegis, Signatures B and E make only a small

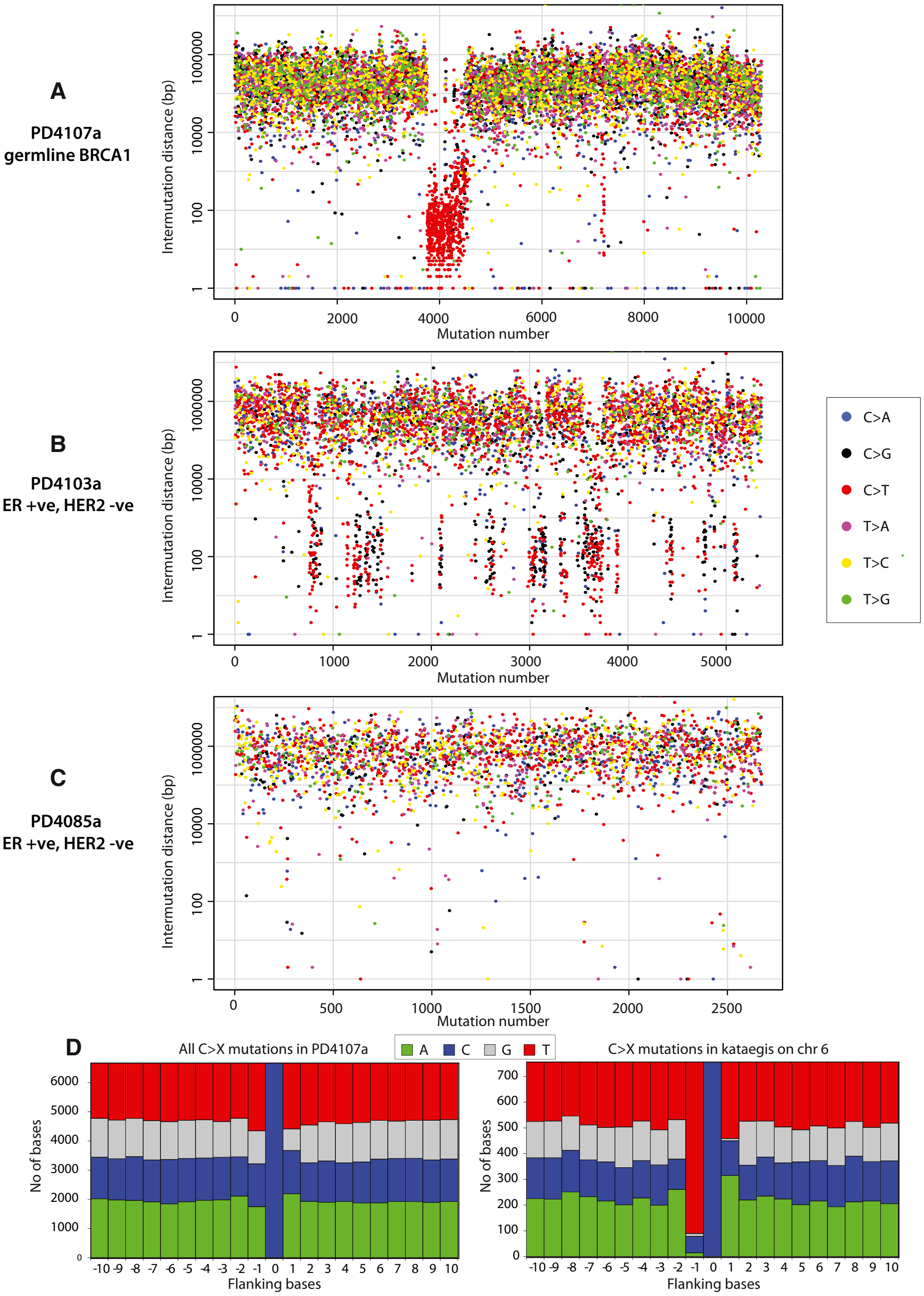

Figure 3. Kataegis, Regional Hypermutation of Base Substitutions, Related to Figure S2

(A) Rainfall plot of PD4107a. Mutations are ordered on the x axis from the first variant on the short arm of chromosome 1 to the last variant on the long arm of chromosome X and are colored according to mutation-type. The distance between each mutation and the one prior to it (the intermutation distance) is plotted on the vertical axis on a log scale. Most mutations in this genome have an intermutation distance of ~10^5 bp to ~10^6 bp. Mutations in a region of hypermutation present as a cluster of lower intermutation distances.

(B) Rainfall plot for PD4103a demonstrating kataegis occurring at multiple loci through the genome.

(C) Rainfall plot for PD4085a, showing no kataegis.

(D) Plots of flanking sequence of all C>X mutations and C>X mutations within the regions of kataegis in PD4107a. Mutated base is at position 0 with ten bases of flanking sequence provided, demonstrating a strong preference for T at the −1 position.

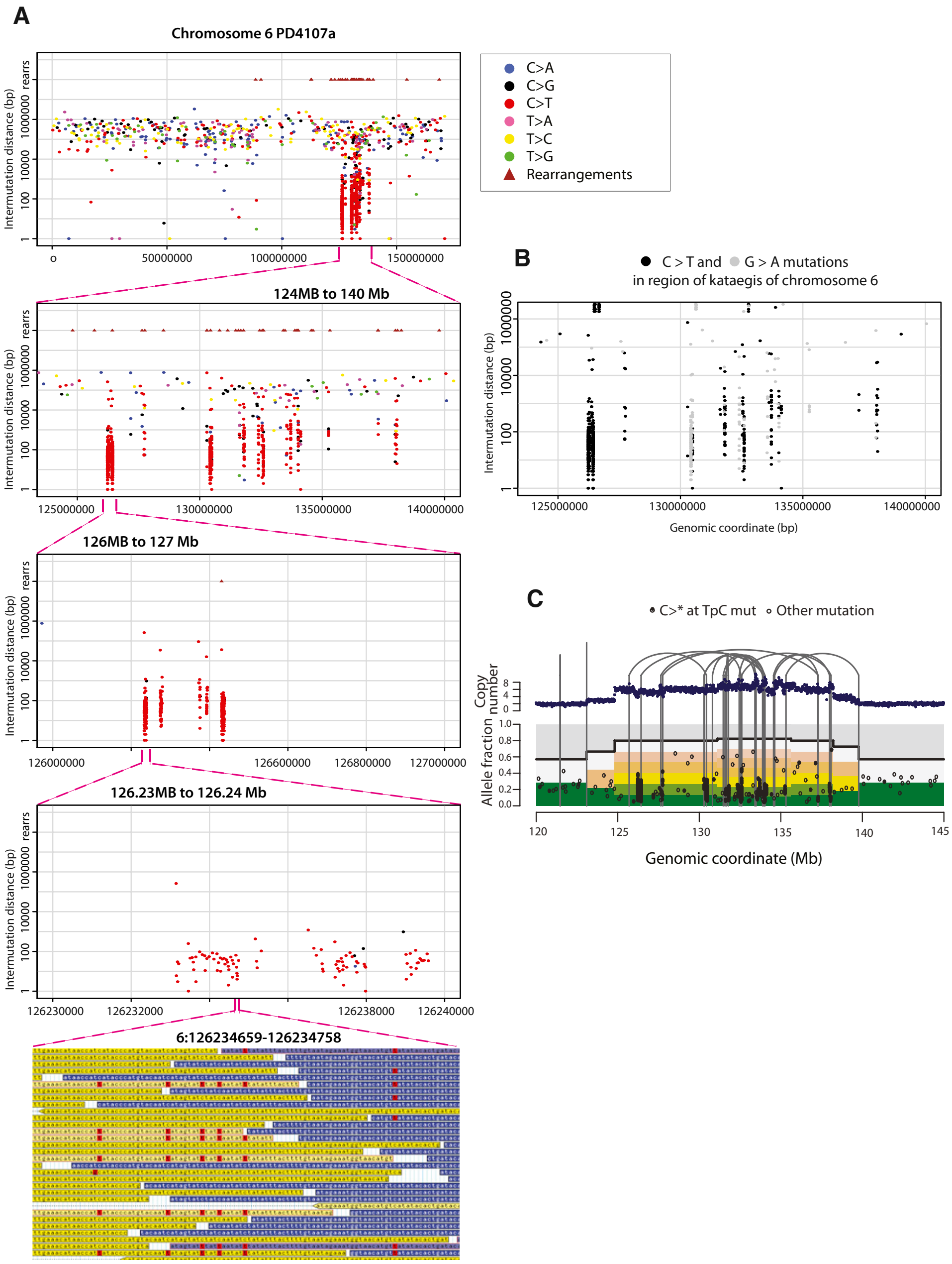

Figure 4. Rainfall Plot for Chromosome 6 of PD4107a

(A) The x axis shows the genomic coordinates of the mutations. Rearrangements are presented as brown triangles (rearrs is an abbreviation for rearrangements). The region of kataegis is highlighted at increasing resolution to demonstrate microclusters within the macrocluster. The processive nature of C>T mutations at TpC context occurring in *cis* is seen in the lowest panel (G-browse image).

(B) Alternating processivity of kataegis in PD4107a. Long regions of C>T mutations are interspersed with regions of G>A mutations.

(C) Kataegis occurs with a variety of rearrangement architectures. Thick top line shows the copy number segments for the region of chromosome 6 of PD4107a. Point mutations are shown in lower panel as black points. x axis reflecting genomic position and y axis represents variant allele fraction. The proportions of reads derived from contaminating normal cells are depicted in gray and the fraction coming from each of the copies of that segment in the tumor cells are depicted by the multiple bars from green to yellow to pink to white. Early mutations will be found relatively higher up these bars, whereas late ones will be seen down the bottom of the variant allele fraction. Grey vertical lines represent rearrangements. Interconnecting lines indicate intrachromosomal rearrangements. On a macroscopic scale, this demonstrates how kataegis can be associated with chromothripsis (within region 130–135 Mb) as well as other rearrangement architectures.

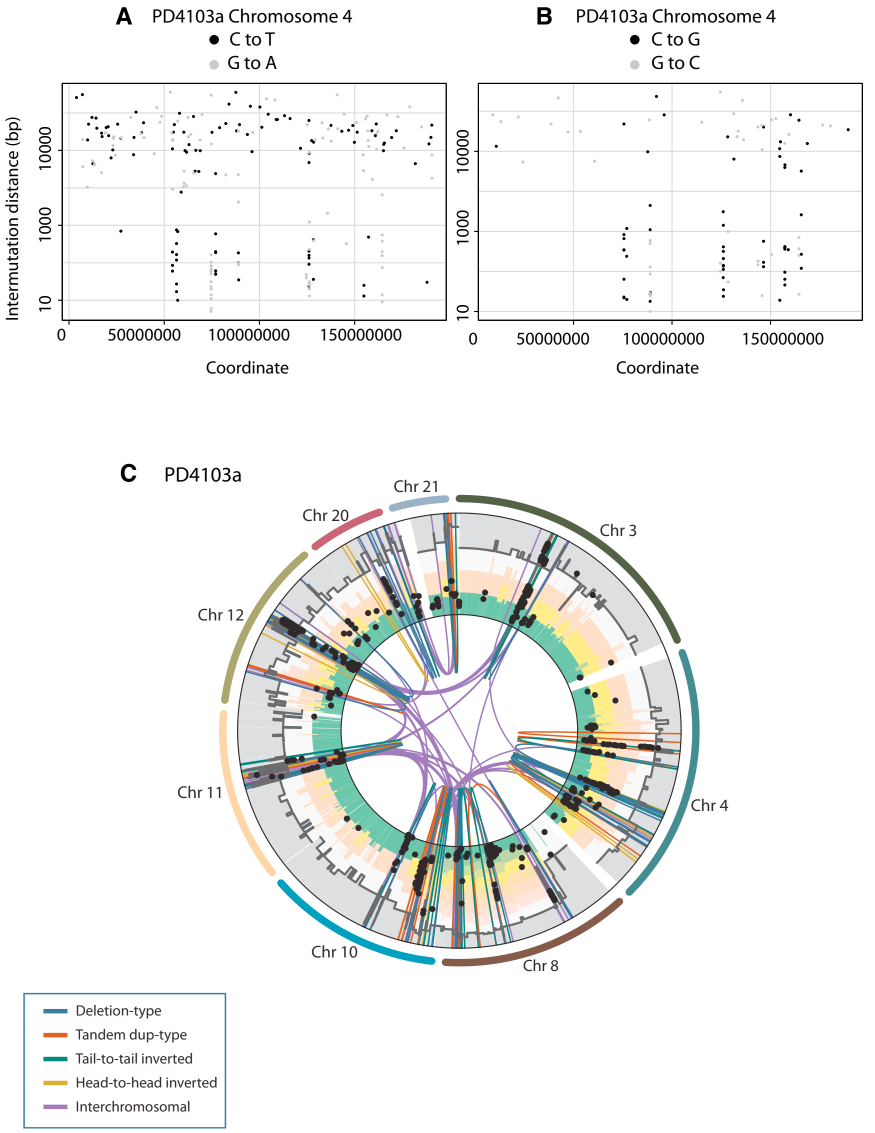

Figure 5. Processivity and Complex Colocalization of Rearrangement Architecture with Kataegis in PD4103a

(A) Stretches of C>T alternate with stretches of G>A on chromosome 4 in PD4103a.

(B) Alternating C>G and G>C mutation on the same chromosome in PD4103a.

(C) The complex web of rearrangements involving 8 chromosomes in PD4103a colocalizing with kataegis.

contribution to the genome-wide mutation catalog. Conversely, Signature B dominates PD4199a (Figures 1A, 1C, and 1D) despite relatively limited kataegis (Figure S2). Therefore, intriguingly, a globally distributed and a localized form of the mutational processes underlying these signatures may exist, and the two forms may operate independently of each other.

The Relationship between Substitution Mutations and Transcription

We next examined the relationship between transcription and prevalence of somatic substitutions. First, we searched for differences in the prevalence of mutations on the transcribed and untranscribed strands (transcriptional strand bias) of protein coding genes. A moderate degree of strand bias was detectable for C>A/G>T transitions across the 21 breast cancer genomes ($p = 1.75 \times 10^{-15}$) and is present in almost all cases (Figure 6A). This bias was characterized by fewer G>T mutations on transcribed than untranscribed strands. A strand bias was also observed for T>G/A>C mutations ($p = 1.5 \times 10^{-4}$) with fewer T>G mutations on transcribed than untranscribed strands. No evidence of a transcriptional strand bias was observed for C>G/G>C, C>T/G>A, T>A/A>T or T>C/A>G mutations.

The best-recognized cause of transcriptional strand bias is TCR that removes nucleotides with bulky adducts from the transcribed strands of genes. Assuming that TCR is responsible for the observed strand biases, the presence of fewer G>T mutations on transcribed than untranscribed strands would suggest that bulky adduct damage to guanine may be the cause of the observed mutations. Similarly, the presence of fewer T>G mutations on transcribed compared to untranscribed strands would suggest that there may have been bulky adduct damage to thymine. The nature of these ubiquitous mutagenic exposures in breast cancer, which may conceivably be of exogenous or endogenous origins, is unknown. However, the hypothesis that TCR is involved is currently unsubstantiated and it may ultimately transpire that other DNA repair, or indeed damage, processes differentially affect the transcribed and untranscribed strands of genes.

We next examined the relationship between levels of gene expression and prevalence of somatic mutation. An inverse correlation between substitution prevalence and gene expression was observed for C>A/G>T ($p = 2.47 \times 10^{-9}$), C>T/G>A ($p = 7.5 \times 10^{-3}$), T>A/A>T ($p = 1.09 \times 10^{-6}$), and T>C/A>G ($p = 1.83 \times 10^{-4}$) mutations for both transcribed and untranscribed strands (Figures 6 and S3A). No correlation was observed for C>G/G>C or T>G/A>C mutations.

The results indicate that mutational processes characterized by both transcriptional strand bias and expression-related mutation prevalence are operative in breast cancer, similar to our previous observations in melanoma and lung cancer. However, T>G/A>C mutations exhibited a transcriptional strand bias but not expression-related mutation prevalence. Conversely,

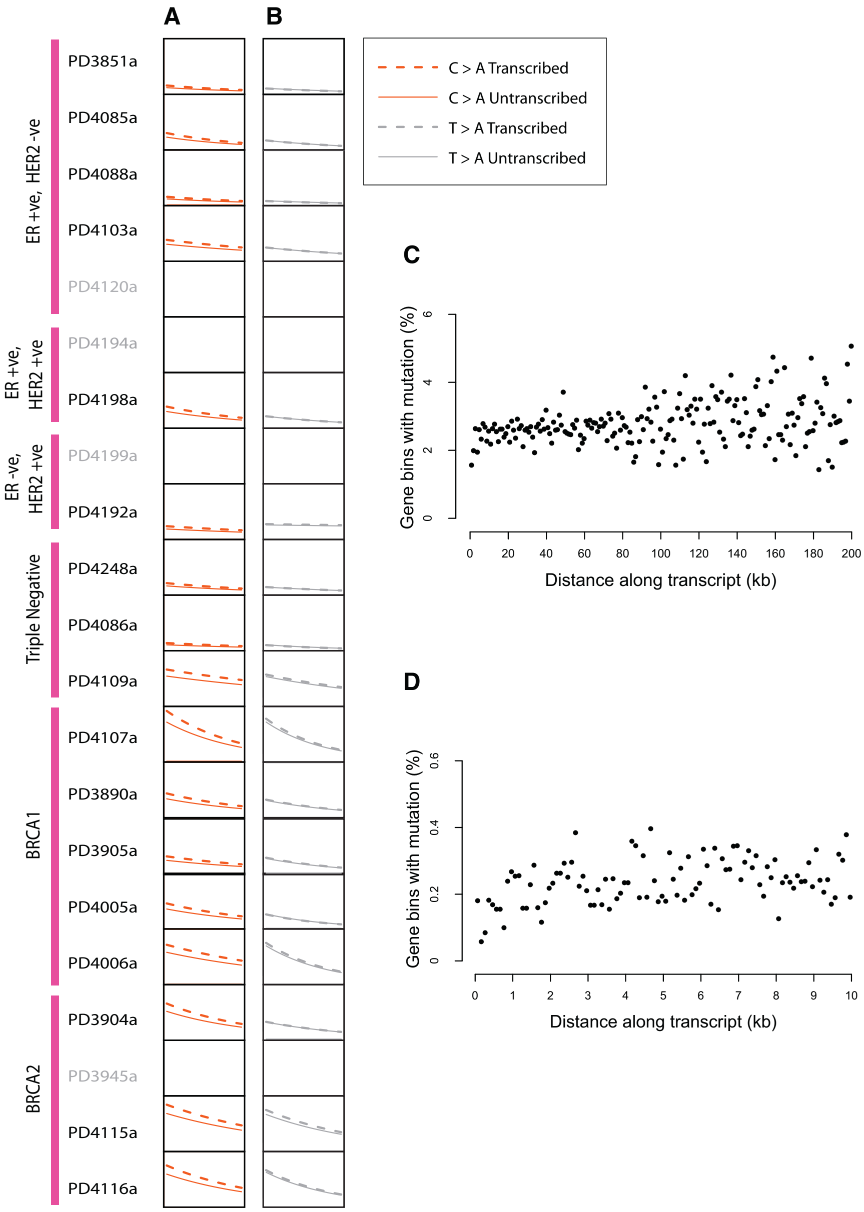

Figure 6. Relationship between Mutation Prevalence and Transcription and/or Expression, Related to Figure S3

Mutation prevalence is expressed as the number of mutations per Mb from 0 to 2 per Mb on the vertical axis. Log 2 expression levels range from 6 to 12 on the horizontal axis. Lines are fitted curves to the data for A and B.

(A) C>A mutations; and (B) T>A mutations. Breast cancer samples without expression data are shown in gray.

(C) Effect of distance from transcription start site on mutation prevalence. Each dot represents a 1 kb bin at increasing distances from all transcription start sites (TSS) up to 200 kb. The y axis shows the percentage of genes in each bin carrying a somatic mutation. The mutation prevalence increases as distance increases from the TSS.

(D) This is particularly marked in the first 1 kb after the TSS. Each dot represents a 100 bp bin.

C>T/G>A, T>A/A>T, and T>C/A>G mutations showed expression-related mutational prevalence but no transcriptional strand bias (Figure 6B and Figure S3A) suggesting that these two features are independent.

Finally, we examined the relationship between distance from the transcriptional start site (TSS) and mutation prevalence in protein coding genes. There was evidence of increasing mutation prevalence at increasing distance from the transcription start site (Figure 6C), suggesting that the suppressive influences of transcription upon mutagenesis described above wane as proximity to the TSS decreases. This effect appears to be particularly pronounced in the first 1 kb from the TSS (Figure 6D). The result confirms the observation previously made on UV light induced C>T mutations in a melanoma.

Microhomology-Mediated Deletions in *BRCA1* and *BRCA2* Mutant Cancers

Of the 2,869 validated somatic indels from the 21 breast cancers, single-base pair indels were the most common in each case (Figure 7A). There was substantial variation in number and pattern of indel, however, with more and larger indels observed in *BRCA1* and *BRCA2* mutant cancers.

The sequences flanking each indel were interrogated for the presence of short tandem repeats or short stretches of identical sequence at the breakpoints (termed overlapping microhomology) (Figure 7B). Repeat-mediated indels were small (1–5 bp), present in all breast cancers, and comprised both deletions and insertions. Microhomology-mediated indels were larger (up to 50bp), mainly deletions and considerably more common in cases with *BRCA1* or *BRCA2* mutations ($p = 2.2 \times 10^{-16}$).

Overlapping microhomology is often considered a signature of nonhomologous end-joining (NHEJ) DNA double-strand break (DSB) repair. The segments of microhomology probably mediate alignment of the two DNA ends that are joined. Because BRCA1 and BRCA2 are involved in homologous recombination (HR)-based DSB repair, the elevated frequency of microhomology-mediated indels in *BRCA1* or *BRCA2* mutant cancers presumably reflects usage of alternative methods of DSB repair in these cancers (Figure 7C).

DISCUSSION

Catalogues of somatic mutation from 21 breast cancers have yielded several insights into underlying mutational mechanisms.

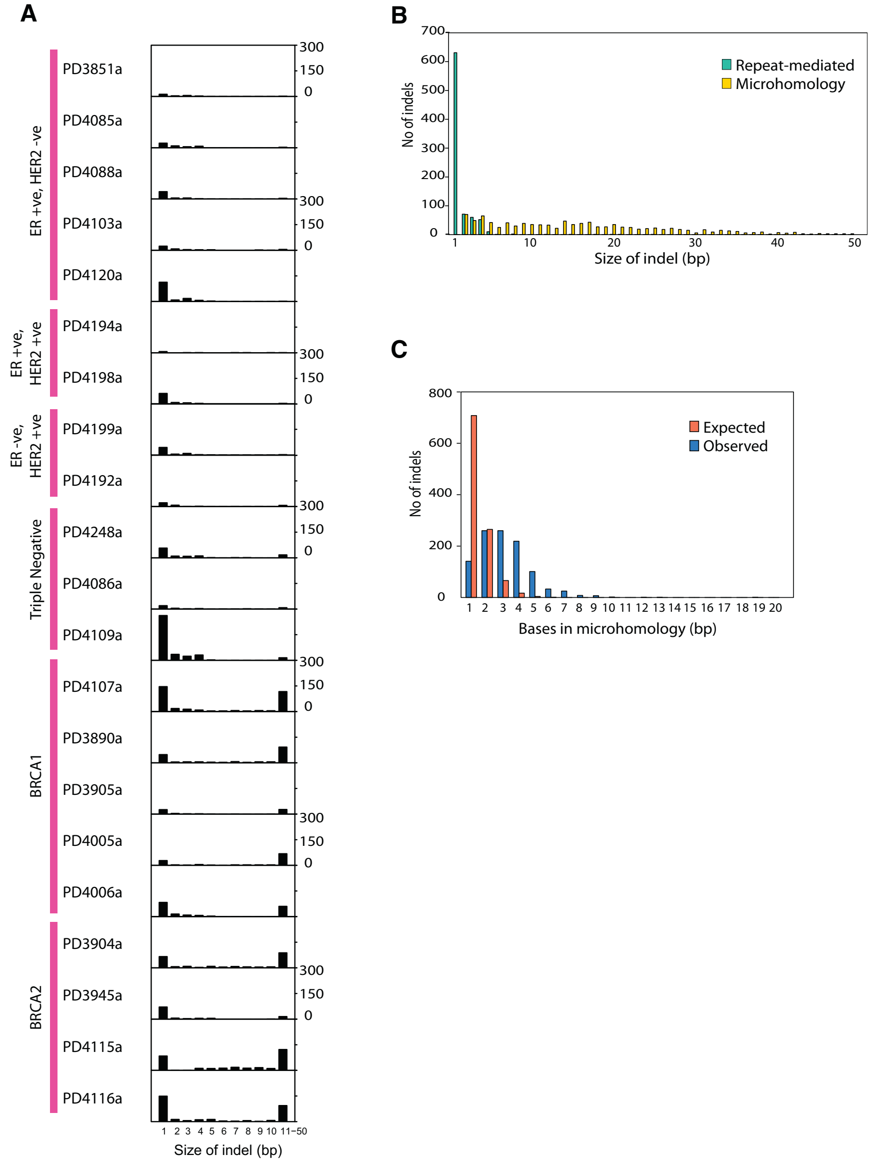

Figure 7. Somatic Mutation Profile of Indels

(A) The x axis shows indel size from 1–10 and all larger indels between 11-50 bp in size grouped in a single bin. The y axis shows the number in each genome from 0–300.

(B) Frequency of indels by indel size. This demonstrates how repeat-mediated indels are usually of smaller size. From a Kolmogorov-Smirnov (K-S) test, the distribution of indel lengths for repeats and microhomologies is significantly different ($p < 2.2 \times 10^{-16}$).

(C) Observed number of bases involved in microhomology at junction of indels versus expected number of bases if microhomology occurred simply by chance.

Five independent single-nucleotide substitution processes appear to have been operating, generating the observed variation in mutation numbers and patterns between cancers. The processes appear to have been acting in combination, either contemporaneously or during different phases of evolution of the cancer clone (Nik-Zainal et al., 2012). Additional subtle processes may exist, and sharper definition of currently characterized processes may follow refinements of NMF and inclusion of other mutational features in the models.

Signature A is likely mediated by deamination of 5-methylcytosine at XpCpG trinucleotides leading to C>T transitions. However, the mechanisms underlying the remainder are currently unknown. Signature B, characterized by C>T, C>G, and C>A substitutions at TpCpX trinucleotides, is responsible for the overwhelming majority of mutations in certain cancer samples and is present in this dominant form in approximately 10% of ER-positive breast cancers (Stephens et al., 2012). The mutational patterns in Signatures C, D, and E have not, to our knowledge, been previously described.

A remarkable process generating regional hypermutation, termed kataegis, is frequently operative in breast cancer. Regional clusters of mutations in cancer have occasionally been observed in experimental models, although not at the mutation density observed here (Wang et al., 2007). Mutations within regions of kataegis bear similarities to those in Signature B, notably the preponderance of C>T and C>G substitutions at TpCpX trinucleotides. Furthermore, they are closely associated with regions of rearrangement and occur on the same chromosome and chromosomal strand over long genomic distances, suggesting that they occur simultaneously or in a processive manner over a short time span (Chen et al., 2011).

On the basis of similarities to mutational patterns observed in other biological contexts or in experimental systems, we propose that the AID/APOBEC family of proteins may be implicated in kataegis and/or in the mutational process underlying Signature B. Although APOBEC1, the founding member of the AID/APOBEC family, was first identified as an RNA-editing enzyme (Teng et al., 1993) several members of the AID/APOBEC family (including APOBEC1 itself) can deaminate cytosine to uracil within DNA, acting as DNA mutators (Harris et al., 2002). AID functions in antibody diversification, deaminating cytosine residues within the immunoglobulin loci in B lymphocytes thereby triggering somatic hypermutation and class-switch recombination (reviewed in Longerich et al., 2006). There are seven APOBEC3 proteins in humans, with the prototype (APOBEC3G) as well as several other APOBEC3s acting on lentiviral replication intermediates constituting an innate pathway of antiretroviral defense (Hultquist et al., 2011; Sheehy et al., 2002).

Although off-target deamination by AID is likely responsible for the mutations and translocations seen in many B cell tumors (reviewed in Nussenzweig and Nussenzweig, 2010), AID is unlikely to account for the mutational signatures described here because it exhibits a strong preference for deaminating C residues flanked by a 5′-purine (Pham et al., 2003). In contrast, the Cs targeted in Signature B and kataegis are preceded by a 5′-T. However, APOBEC1 (when acting on DNA) and the APOBEC3 enzymes (apart from APOBEC3G) favor C residues flanked by a 5′-T (Harris et al., 2002; Hultquist et al., 2011). Furthermore, transgenic overexpression of APOBEC1 is associated with cancer development (Yamanaka et al., 1995) and enforced overexpression of APOBEC3A causes genomic damage and mutation (Landry et al., 2011; Stenglein et al., 2010; Suspène et al., 2011). Thus APOBEC1 and some APOBEC3s are attractive candidates for the mechanisms underlying kataegis and/or mutation Signature B. Thus far, we have not observed a clear correlation between overexpression of a member of the AID/APOBEC family and kataegis or Signature B, although some key samples lack expression data.

Signature E also exhibits mutations at TpCpX trinucleotides, but is characterized by a much lower fraction of C>T mutations than Signature B. It is possible that both result from cytidine to uracil deamination by an APOBEC family member, but that the different signatures are sequelae of different repair mechanisms following the deamination step. C>T transitions may simply result from DNA replication across uracil. However, if uracil is excised by uracil-DNA glycosylase (UNG) as part of base excision repair (BER), an abasic site is generated (Wilson and Bohr, 2007). The partiality for C>G transversions in Signature E may reflect preferential insertion of cytosine opposite such an UNG-mediated abasic site. The propensity to introduce cytosine opposite an abasic site is characteristic of REV1 translesion polymerase, which is known to function in BER (Jansen et al., 2006; Ross and Sale, 2006). Thus, Signature B may be caused by a combination of replication and BER, whereas Signature E may be the imprint of the almost exclusive activity of BER on uracil.

Further studies are required to explore whether and how AID/APOBEC family members contribute to mutagenesis in cancer. If they are implicated in kataegis, current understanding of the mode of action of AID in immunoglubulin gene somatic hypermutation and class switch recombination would suggest that their primary effect is through deamination of cytidine to uracil, with substitutions and rearrangements both consequent upon this initiating event. If so, an important remaining question is how the activity of the enzymes is targeted to the regions of kataegis. Furthermore, if the same enzymes from the AID/APOBEC family are also involved in mutation Signatures B and possibly E, it remains to be understood how their activities can be unleashed upon the whole genome without apparent relation to the presence of rearrangements, as opposed to being regionally targeted in the vicinity of rearrangements in kataegis.

Other mechanisms and enzymatic activities may, however, be responsible for kataegis. If so, the question of which constitutes the primary set of lesions, the rearrangements, or the substitutions observed in kataegis, remains to be addressed. If a stochastic event in a cell nucleus results in a DNA DSB and repair of this break is associated with accumulation of substitutions in the vicinity of the consequent rearrangement, this could provide an explanation for the regional targeting of kataegis. Indeed, such mechanisms have been reported in yeast (Deem et al., 2011; Hicks et al., 2010).

In all the breast cancers, double-nucleotide substitutions were much more common than expected by chance adjacency of single-nucleotide substitutions, suggesting the existence of one or more biological processes responsible for their presence. Currently, the best-characterized double-nucleotide substitutions in human cancer are the CpC>TpT mutations found in skin tumors, which are generally attributed to the pyrimidine dimers generated by UV light exposure. In principle, the dinucleotide mutations observed in breast cancer could also be due to exposures with a propensity to damage adjacent DNA bases. However, other mechanisms are also plausible, for example error prone polymerases that have a higher risk of misincorporation at a base adjacent to one that is damaged.

BRCA1 and BRCA2 are implicated in HR-based DNA repair processes. The distinctive profile of small deletions with rearrangement breakpoints showing overlapping microhomology in *BRCA1* and *BRCA2* mutant cancers is therefore compatible with these processes being defective and of NHEJ or other error-prone mechanisms of DSB repair acting in their place. Interestingly, the combinations of base substitution signatures in *BRCA1* and *BRCA2* mutant cases are also similar. These similarities in mutational signatures contrast strikingly with differences in histology and gene expression profiles between *BRCA1* and *BRCA2* mutant cancers (Hedenfalk et al., 2001; Palacios et al., 2008; Perou et al., 2000; Sørlie et al., 2001). Mutational patterns, which are probably more closely related to the underlying biological defect, therefore appear to report similarities in disease pathogenesis between *BRCA1* and *BRCA2* mutant cancers better than cellular phenotype. *BRCA1* and *BRCA2* cancers are particularly responsive to some DNA damaging agents and inhibitors of DNA repair, notably PARP inhibitors (Fong et al., 2009). Because some breast cancers without mutations in *BRCA1* and *BRCA2* are reported to respond to these treatments (Forster et al., 2011) it will be interesting to explore whether mutational patterns characteristic of *BRCA1* and *BRCA2* null cancers are better predictors of response to these therapies than the presence of mutations in the two genes.

These 21 genomes have yielded further evidence of complex relationships between mutagenesis and transcription. A transcriptional strand bias was found for C>A/G>T mutations in most of the cancers. If TCR is responsible, DNA damage by bulky adducts may be implicated in breast cancer pathogenesis. In principle, these could result from exogenous exposures. Indeed, many carcinogens cause adducts on guanine. Alternatively, an exposure could be endogenous in origin, for example reactive oxygen species (Hori et al., 2011) or intermediates of oxidative estrogen metabolism (Spencer et al., 2012). Both can cause damage to guanine and, although preferentially repaired by BER, some lesions can be substrates for TCR (Hanawalt and Spivak, 2008). If TCR is not involved, then the data suggest that other uncharacterized forms of transcription coupled DNA damage or repair exist.

The relationship between gene expression levels and mutation prevalence, previously reported in a malignant melanoma and a small cell lung cancer (Pleasance et al., 2010a; Pleasance et al., 2010b), has been extended here to primary breast cancers. The relationship is again inverse in nature, with more somatic substitutions in poorly expressed genes. The phenomenon could, in principle, be due to an increased sensitivity to DNA damage and/or less efficient repair in poorly expressed genes. The fact that it applies to the untranscribed strands of genes and does not correlate with the presence of transcriptional strand bias suggests that these have different underlying mechanisms. One possibility is that the genome-wide form of NER is recruited more effectively to highly transcribed genes.

This study has started to untangle and characterize the mutational processes that contribute to breast cancer. The data are derived from only 21 genomes and similar analyses of thousands of cancers by the International Cancer Genome Consortium (Hudson et al., 2010) will likely yield evidence of further mutational processes and better definition of those already known. Nevertheless, the analyses have provided a level of characterization of mutational processes in cancer that was previously impossible and illustrate the power of whole cancer genome sequences, yielding essentially complete catalogs of somatic mutations, to further understanding of mechanisms of DNA damage and repair.

EXPERIMENTAL PROCEDURES

Samples and Massively Parallel Sequencing

DNA was extracted from 21 breast cancers and normal tissue from the same individuals. Short insert 500 bp library construction, flowcell preparation and cluster generation were according to the Illumina no-PCR library protocol (Kozarewa et al., 2009). 108 base or 100 base paired-end sequencing were performed on Illumina GAIIx or Hiseq 2000 genome analyzers respectively, as described in the Illumina Genome Analyzer operating manual.

Short insert 2*108 bp or 2*100 bp paired-end reads were aligned to the reference human genome (NCBI37) by using BWA (Li and Durbin, 2009). Genome sequence data have been deposited at the European Genome-Phenome Archive (http://www.ebi.ac.uk/ega/ at the EBI) with accession number EGAD00001000138. SNP6 array data have been deposited with ArrayExpress Archive (EBI, accession number E-MTAB-1087).

Mutation-Calling

An in-house bespoke algorithm, CaVEMan was used for calling somatic substitutions. Insertions and deletions in the tumor and normal genomes were called by using a modified Pindel version 0.2.0 on the NCBI37 genome build (Ye et al., 2009). Postprocessing filters were developed to improve the specificity of mutation-calling. Structural variants were called from the short insert data by using MAQ alignments as previously described (Campbell et al., 2008; Stephens et al., 2009). Structural variants in association with copy number segments were sought to improve sensitivity of detection. Tumor DNA samples were analyzed by Affymetrix SNP6 microarrays (Bignell et al., 2010). Copy number and allelic ratio profiles were statistically processed by using the ASCAT algorithm, version 2.0 (Van Loo et al., 2010). Validation of substitutions and indels was performed by Roche 454 pyrosequencing or capillary sequencing. Structural variants were confirmed by custom-designed PCR across the rearrangement breakpoint or by local sequence assembly. All confirmations were performed in both tumor and normal. Gene expression data were derived from the Illumina Human HT12 Expression BeadChip array, and processed as previously described (Pleasance et al., 2010a). Somatic mutation data are available via hyperlinks in Table S1B and are also available via COSMIC at http://www.sanger.ac.uk/genetics/CGP/cosmic/ and have been annotated to Ensembl v58.

Statistical Analysis

Mutational processes were extracted by using nonnegative matrix factorization. Monte Carlo simulations were performed to assess how randomly distributed mutations differed to the primary cancer genomes. A Kolmogorov-Smirnov test was used to compare the distribution of indels mediated by repeats or microhomology.

ACCESSION NUMBERS

The European Genome-Phenome Archive accession number for the sequence reported in this paper is EGAD00001000138. The ArrayExpress Archive accession number for the SNP6 array data reported in this paper is E-MTAB-1087.

SUPPLEMENTAL INFORMATION

Supplemental Information includes Extended Experimental Procedures, three figures, and one table and can be found with this article online at doi:10.1016/j.cell.2012.04.024.

ACKNOWLEDGMENTS

This work was supported by the Wellcome Trust (grant reference 098051) and Breakthrough Breast Cancer Research (ICGC 08/09). Individual support: S.N.Z. (Wellcome Trust Clinical Research Training Fellow); P.J.C. (Wellcome Trust Senior Clinical Research Fellow grant reference WT088340MA); P.V.L. (Research Foundation - Flanders [FWO]); A.S. (National Research Council Canada [HL Holmes Award] and EMBO); I.V. (International Human Frontier Science Program Organization); J.W.M.M. (Netherlands Genomics Initiative (NGI)/Netherlands Organization for Scientific Research); A.L.B.D. and A.L. (Norwegian Research Council, The Norwegian Cancer Society, The Radium Hospital Foundation and Health Region South-East); S.M (European Community's Seventh Framework Programme [FP7/2010-2014] grant reference 242006 Breast Cancer Somatic Genetics Study [BASIS]). We thank Nancy Miller for her activities related to the curation and collection of the clinical data associated with this study. We acknowledge the Core Sequencing Facility and IT groups of the Wellcome Trust Sanger Institute, the Breakthrough Breast Cancer Unit, members of the ICGC Breast Cancer Working Group, the OSBREAC Consortium of Oslo University Hospital, the United States National Cancer Institute Specialized Program Of Research Excellence (grant reference CA089393) and Dr. Peter Watson of the BCCA Tumour Tissue Repository and the Centre for Translational Genomics (CTAG). The authors acknowledge financial support from the Department of Health via NIHR comprehensive Biomedical Research Centre award to Guy's and St Thomas' NHS Foundation Trust, King's College London, and King's College Hospital NHS Foundation Trust. We thank George Vassiliou for pointing us to the term "kataegis." J.E.G. is a consultant for Generation Health.

Received: December 14, 2011
Revised: March 12, 2012
Accepted: April 30, 2012
Published online: May 17, 2012

REFERENCES

Berger, M.F., Lawrence, M.S., Demichelis, F., Drier, Y., Cibulskis, K., Sivachenko, A.Y., Sboner, A., Esgueva, R., Pflueger, D., Sougnez, C., et al. (2011). The genomic complexity of primary human prostate cancer. Nature *470*, 214–220.

Berry, M.W., Browne, M., Langville, A.N., Pauca, V.P., and Plemmons, R.J. (2007). Algorithms and applications for approximate nonnegative matrix factorization. Comput. Stat. Data Anal. *52*, 155–173.

Bignell, G.R., Greenman, C.D., Davies, H., Butler, A.P., Edkins, S., Andrews, J.M., Buck, G., Chen, L., Beare, D., Latimer, C., et al. (2010). Signatures of mutation and selection in the cancer genome. Nature *463*, 893–898.

Campbell, P.J., Stephens, P.J., Pleasance, E.D., O'Meara, S., Li, H., Santarius, T., Stebbings, L.A., Leroy, C., Edkins, S., Hardy, C., et al. (2008). Identification of somatically acquired rearrangements in cancer using genome-wide massively parallel paired-end sequencing. Nat. Genet. *40*, 722–729.

Chapman, M.A., Lawrence, M.S., Keats, J.J., Cibulskis, K., Sougnez, C., Schinzel, A.C., Harview, C.L., Brunet, J.P., Ahmann, G.J., Adli, M., et al. (2011). Initial genome sequencing and analysis of multiple myeloma. Nature *471*, 467–472.

Chen, J.M., Férec, C., and Cooper, D.N. (2011). Transient hypermutability, chromothripsis and replication-based mechanisms in the generation of concurrent clustered mutations. Mutat. Res. *750*, 52–59.

Deem, A., Keszthelyi, A., Blackgrove, T., Vayl, A., Coffey, B., Mathur, R., Chabes, A., and Malkova, A. (2011). Break-induced replication is highly inaccurate. PLoS Biol. *9*, e1000594.

Ding, L., Ellis, M.J., Li, S., Larson, D.E., Chen, K., Wallis, J.W., Harris, C.C., McLellan, M.D., Fulton, R.S., Fulton, L.L., et al. (2010). Genome remodelling in a basal-like breast cancer metastasis and xenograft. Nature *464*, 999–1005.

Fong, P.C., Boss, D.S., Yap, T.A., Tutt, A., Wu, P., Mergui-Roelvink, M., Mortimer, P., Swaisland, H., Lau, A., O'Connor, M.J., et al. (2009). Inhibition of poly(ADP-ribose) polymerase in tumors from BRCA mutation carriers. N. Engl. J. Med. *361*, 123–134.

Forster, M.D., Dedes, K.J., Sandhu, S., Frentzas, S., Kristeleit, R., Ashworth, A., Poole, C.J., Weigelt, B., Kaye, S.B., and Molife, L.R. (2011). Treatment with olaparib in a patient with PTEN-deficient endometrioid endometrial cancer. Nature reviews. Clin. Oncol. *8*, 302–306.

Greenman, C., Stephens, P., Smith, R., Dalgliesh, G.L., Hunter, C., Bignell, G., Davies, H., Teague, J., Butler, A., Stevens, C., et al. (2007). Patterns of somatic mutation in human cancer genomes. Nature *446*, 153–158.

Hainaut, P., and Pfeifer, G.P. (2001). Patterns of p53 G→T transversions in lung cancers reflect the primary mutagenic signature of DNA-damage by tobacco smoke. Carcinogenesis *22*, 367–374.

Hanawalt, P.C., and Spivak, G. (2008). Transcription-coupled DNA repair: two decades of progress and surprises. Nat. Rev. Mol. Cell Biol. *9*, 958–970.

Harris, R.S., Petersen-Mahrt, S.K., and Neuberger, M.S. (2002). RNA editing enzyme APOBEC1 and some of its homologs can act as DNA mutators. Mol. Cell *10*, 1247–1253.

Hedenfalk, I., Duggan, D., Chen, Y., Radmacher, M., Bittner, M., Simon, R., Meltzer, P., Gusterson, B., Esteller, M., Kallioniemi, O.P., et al. (2001). Gene-expression profiles in hereditary breast cancer. N. Engl. J. Med. *344*, 539–548.

Hicks, W.M., Kim, M., and Haber, J.E. (2010). Increased mutagenesis and unique mutation signature associated with mitotic gene conversion. Science *329*, 82–85.

Hori, M., Suzuki, T., Minakawa, N., Matsuda, A., Harashima, H., and Kamiya, H. (2011). Mutagenicity of secondary oxidation products of 8-oxo-7,8-dihydro-2′-deoxyguanosine 5′-triphosphate (8-hydroxy-2′- deoxyguanosine 5′-triphosphate). Mutat. Res. *714*, 11–16.

Hudson, T.J., Anderson, W., Artez, A., Barker, A.D., Bell, C., Bernabé, R.R., Bhan, M.K., Calvo, F., Eerola, I., Gerhard, D.S., et al; International Cancer Genome Consortium. (2010). International network of cancer genome projects. Nature *464*, 993–998.

Hultquist, J.F., Lengyel, J.A., Refsland, E.W., LaRue, R.S., Lackey, L., Brown, W.L., and Harris, R.S. (2011). Human and rhesus APOBEC3D, APOBEC3F, APOBEC3G, and APOBEC3H demonstrate a conserved capacity to restrict Vif-deficient HIV-1. J. Virol. *85*, 11220–11234.

Jansen, J.G., Langerak, P., Tsaalbi-Shtylik, A., van den Berk, P., Jacobs, H., and de Wind, N. (2006). Strand-biased defect in C/G transversions in hypermutating immunoglobulin genes in Rev1-deficient mice. J. Exp. Med. *203*, 319–323.

Kozarewa, I., Ning, Z., Quail, M.A., Sanders, M.J., Berriman, M., and Turner, D.J. (2009). Amplification-free Illumina sequencing-library preparation facilitates improved mapping and assembly of (G+C)-biased genomes. Nat. Methods *6*, 291–295.

Landry, S., Narvaiza, I., Linfesty, D.C., and Weitzman, M.D. (2011). APOBEC3A can activate the DNA damage response and cause cell-cycle arrest. EMBO Rep. *12*, 444–450.

Lee, D.D., and Seung, H.S. (1999). Learning the parts of objects by non-negative matrix factorization. Nature *401*, 788–791.

Lee, W., Jiang, Z., Liu, J., Haverty, P.M., Guan, Y., Stinson, J., Yue, P., Zhang, Y., Pant, K.P., Bhatt, D., et al. (2010). The mutation spectrum revealed by paired genome sequences from a lung cancer patient. Nature *465*, 473–477.

Ley, T.J., Mardis, E.R., Ding, L., Fulton, B., McLellan, M.D., Chen, K., Dooling, D., Dunford-Shore, B.H., McGrath, S., Hickenbotham, M., et al. (2008). DNA sequencing of a cytogenetically normal acute myeloid leukaemia genome. Nature *456*, 66–72.

Li, H., and Durbin, R. (2009). Fast and accurate short read alignment with Burrows-Wheeler transform. Bioinformatics *25*, 1754–1760.

Longerich, S., Basu, U., Alt, F., and Storb, U. (2006). AID in somatic hypermutation and class switch recombination. Curr. Opin. Immunol. *18*, 164–174.

Macé, K., Aguilar, F., Wang, J.S., Vautravers, P., Gómez-Lechón, M., Gonzalez, F.J., Groopman, J., Harris, C.C., and Pfeifer, A.M. (1997). Aflatoxin B1-induced DNA adduct formation and p53 mutations in CYP450-expressing human liver cell lines. Carcinogenesis *18*, 1291–1297.

Mardis, E.R., Ding, L., Dooling, D.J., Larson, D.E., McLellan, M.D., Chen, K., Koboldt, D.C., Fulton, R.S., Delehaunty, K.D., McGrath, S.D., et al. (2009). Recurring mutations found by sequencing an acute myeloid leukemia genome. N. Engl. J. Med. *361*, 1058–1066.

Nedelko, T., Arlt, V.M., Phillips, D.H., and Hollstein, M. (2009). TP53 mutation signature supports involvement of aristolochic acid in the aetiology of endemic nephropathy-associated tumours. Int. J. Cancer *124*, 987–990.

Nik-Zainal, S., Van Loo, P., Wedge, D.C., Alexandrov, L.B., Greenman, C.D., Lau, K.W., Raine, K., Jones, D., Marshall, J., Ramakrishna, M., et al. (2012). The life history of 21 breast cancers. Cell *149*. Published online May 17, 2012. 10.1016/j.cell.2012.04.023.

Nussenzweig, A., and Nussenzweig, M.C. (2010). Origin of chromosomal translocations in lymphoid cancer. Cell *141*, 27–38.

Palacios, J., Robles-Frías, M.J., Castilla, M.A., López-García, M.A., and Benítez, J. (2008). The molecular pathology of hereditary breast cancer. Pathobiology *75*, 85–94.

Perou, C.M., Sørlie, T., Eisen, M.B., van de Rijn, M., Jeffrey, S.S., Rees, C.A., Pollack, J.R., Ross, D.T., Johnsen, H., Akslen, L.A., et al. (2000). Molecular portraits of human breast tumours. Nature *406*, 747–752.

Pfeifer, G.P., Denissenko, M.F., Olivier, M., Tretyakova, N., Hecht, S.S., and Hainaut, P. (2002). Tobacco smoke carcinogens, DNA damage and p53 mutations in smoking-associated cancers. Oncogene *21*, 7435–7451.

Pfeifer, G.P., You, Y.H., and Besaratinia, A. (2005). Mutations induced by ultraviolet light. Mutat. Res. *571*, 19–31.

Pham, P., Bransteitter, R., Petruska, J., and Goodman, M.F. (2003). Processive AID-catalysed cytosine deamination on single-stranded DNA simulates somatic hypermutation. Nature *424*, 103–107.

Pleasance, E.D., Cheetham, R.K., Stephens, P.J., McBride, D.J., Humphray, S.J., Greenman, C.D., Varela, I., Lin, M.L., Ordóñez, G.R., Bignell, G.R., et al. (2010a). A comprehensive catalogue of somatic mutations from a human cancer genome. Nature *463*, 191–196.

Pleasance, E.D., Stephens, P.J., O'Meara, S., McBride, D.J., Meynert, A., Jones, D., Lin, M.L., Beare, D., Lau, K.W., Greenman, C., et al. (2010b). A small-cell lung cancer genome with complex signatures of tobacco exposure. Nature *463*, 184–190.

Ross, A.L., and Sale, J.E. (2006). The catalytic activity of REV1 is employed during immunoglobulin gene diversification in DT40. Mol. Immunol. *43*, 1587–1594.

Shah, S.P., Morin, R.D., Khattra, J., Prentice, L., Pugh, T., Burleigh, A., Delaney, A., Gelmon, K., Guliany, R., Senz, J., et al. (2009). Mutational

evolution in a lobular breast tumour profiled at single nucleotide resolution. Nature *461*, 809–813.

Sheehy, A.M., Gaddis, N.C., Choi, J.D., and Malim, M.H. (2002). Isolation of a human gene that inhibits HIV-1 infection and is suppressed by the viral Vif protein. Nature *418*, 646–650.

Sørlie, T., Perou, C.M., Tibshirani, R., Aas, T., Geisler, S., Johnsen, H., Hastie, T., Eisen, M.B., van de Rijn, M., Jeffrey, S.S., et al. (2001). Gene expression patterns of breast carcinomas distinguish tumor subclasses with clinical implications. Proc. Natl. Acad. Sci. USA *98*, 10869–10874.

Spencer, W.A., Vadhanam, M.V., Jeyabalan, J., and Gupta, R.C. (2012). Oxidative DNA damage following microsome/Cu(II)-mediated activation of the estrogens, 17β-estradiol, equilenin, and equilin: role of reactive oxygen species. Chem. Res. Toxicol. *25*, 305–314.

Stenglein, M.D., Burns, M.B., Li, M., Lengyel, J., and Harris, R.S. (2010). APOBEC3 proteins mediate the clearance of foreign DNA from human cells. Nat. Struct. Mol. Biol. *17*, 222–229.

Stephens, P., Edkins, S., Davies, H., Greenman, C., Cox, C., Hunter, C., Bignell, G., Teague, J., Smith, R., Stevens, C., et al. (2005). A screen of the complete protein kinase gene family identifies diverse patterns of somatic mutations in human breast cancer. Nat. Genet. *37*, 590–592.

Stephens, P.J., McBride, D.J., Lin, M.L., Varela, I., Pleasance, E.D., Simpson, J.T., Stebbings, L.A., Leroy, C., Edkins, S., Mudie, L.J., et al. (2009). Complex landscapes of somatic rearrangement in human breast cancer genomes. Nature *462*, 1005–1010.

Stephens, P.J., Greenman, C.D., Fu, B., Yang, F., Bignell, G.R., Mudie, L.J., Pleasance, E.D., Lau, K.W., Beare, D., Stebbings, L.A., et al. (2011). Massive genomic rearrangement acquired in a single catastrophic event during cancer development. Cell *144*, 27–40.

Stephens, P.J., Tarpey, P., Davies, H., Van Loo, P., Greenman, C., Varela, I., Nik Zainal, S., Bignell, G.R., Martin, S., Wedge, D.C., et al. (2012). The landscape of cancer genes and mutational processes in breast cancer. Nature *485*, in press. Published online May 17, 2012. 10.1038/nature11017.

Stratton, M.R. (2011). Exploring the genomes of cancer cells: progress and promise. Science *331*, 1553–1558.

Stratton, M.R., Campbell, P.J., and Futreal, P.A. (2009). The cancer genome. Nature *458*, 719–724.

Suspène, R., Aynaud, M.M., Guétard, D., Henry, M., Eckhoff, G., Marchio, A., Pineau, P., Dejean, A., Vartanian, J.P., and Wain-Hobson, S. (2011). Somatic hypermutation of human mitochondrial and nuclear DNA by APOBEC3 cytidine deaminases, a pathway for DNA catabolism. Proc. Natl. Acad. Sci. USA *108*, 4858–4863.

Tao, Y., Ruan, J., Yeh, S.H., Lu, X., Wang, Y., Zhai, W., Cai, J., Ling, S., Gong, Q., Chong, Z., et al. (2011). Rapid growth of a hepatocellular carcinoma and the driving mutations revealed by cell-population genetic analysis of whole-genome data. Proc. Natl. Acad. Sci. USA *108*, 12042–12047.

Teng, B., Burant, C.F., and Davidson, N.O. (1993). Molecular cloning of an apolipoprotein B messenger RNA editing protein. Science *260*, 1816–1819.

Van Loo, P., Nordgard, S.H., Lingjærde, O.C., Russnes, H.G., Rye, I.H., Sun, W., Weigman, V.J., Marynen, P., Zetterberg, A., Naume, B., et al. (2010). Allele-specific copy number analysis of tumors. Proc. Natl. Acad. Sci. USA *107*, 16910–16915.

Wang, J., Gonzalez, K.D., Scaringe, W.A., Tsai, K., Liu, N., Gu, D., Li, W., Hill, K.A., and Sommer, S.S. (2007). Evidence for mutation showers. Proc. Natl. Acad. Sci. USA *104*, 8403–8408.

Waters, T.R., and Swann, P.F. (2000). Thymine-DNA glycosylase and G to A transition mutations at CpG sites. Mutat. Res. *462*, 137–147.

Wilson, D.M., 3rd, and Bohr, V.A. (2007). The mechanics of base excision repair, and its relationship to aging and disease. DNA Repair (Amst.) *6*, 544–559.

Yamanaka, S., Balestra, M.E., Ferrell, L.D., Fan, J., Arnold, K.S., Taylor, S., Taylor, J.M., and Innerarity, T.L. (1995). Apolipoprotein B mRNA-editing protein induces hepatocellular carcinoma and dysplasia in transgenic animals. Proc. Natl. Acad. Sci. USA *92*, 8483–8487.

Ye, K., Schulz, M.H., Long, Q., Apweiler, R., and Ning, Z. (2009). Pindel: a pattern growth approach to detect break points of large deletions and medium sized insertions from paired-end short reads. Bioinformatics *25*, 2865–2871.

EXTENDED EXPERIMENTAL PROCEDURES

Samples and Massively Parallel Sequencing

DNA was extracted from 21 breast cancers as well as matched normal tissue derived from the same individuals. Samples had previously been subjected to pathology review and only samples assessed as being composed of > 70% tumor cells, were accepted for these analyses.

Short insert 500bp library construction, flowcell preparation and cluster generation was performed according to the Illumina no-PCR library protocol (Kozarewa et al., 2009). 108 base paired-end sequencing or 100 base paired-end sequencing was performed on Illumina GAIIx genome analysers or Illumina Hiseq 2000 analysers respectively, as described in the Illumina Genome Analyzer operating manual.

Short insert 2*108 bp or 2*100 bp paired-end reads were aligned to the reference human genome (NCBI37) by using BWA (Li and Durbin, 2009). An average of 30-fold sequence coverage was required for both tumor and normal genomes. One breast cancer, PD4120a, was sequenced to ~188-fold coverage (Table S1A).

Mutation-Calling: Substitutions

An in-house bespoke algorithm, CaVEMan (Cancer Variants Through Expectation Maximization) was used for calling somatic substitutions. In brief, CaVEMan is a somatic base substitution caller that utilizes an expectation maximization (EM) algorithm and is designed for calling variants in new sequencing technology reads. Given the reference base, copy number status and fraction of aberrant tumor cells present in each cancer sample, CaVEMan generates a probability score for potential genotypes at each genomic position. A putative 'somatic' genotype probability of 95% and above was applied as a cut off. A high specificity was essential for the nature of downstream analyses applied in this study. As such, further postprocessing filters of potential 'somatic' genotypes were designed to eliminate false positive calls arising from:

- genomic features that generate mapping errors e.g., regions of excessively high coverage due to collapsed repeat sequences in the reference genome (http://genome.ucsc.edu/)
- systematic sequencing artifacts e.g., motifs known to cause errors of phasing during the sequencing reaction or sequencing artifacts arising in at least 5% of at least 2 samples from a panel of normal samples
- germline insertions/deletions.

1%–5% of putative somatic substitution variants from each cancer genome were sampled for validation in order to make an assessment of the specificity of these cancer genomes.

Mutation-Calling: Insertions/Deletions

Insertions and deletions in the tumor and normal genomes were called by using a modified Pindel version 0.2.0 on the NCBI37 genome build (Ye et al., 2009).

Indels were required to be present in 5 reads or more in the tumor and not present in the matched normal sample. Variants were also screened against a panel of normal samples and were excluded if present in at least 5% of reads in at least 2 samples from this panel. All indels reported in this study have been validated.

Mutation-Calling: Copy Number

Tumor DNA samples were analyzed by Affymetrix SNP6 microarrays (Bignell et al., 2010). Copy number and allelic ratio profiles were statistically processed by using the ASCAT algorithm, version 2.0 (Van Loo et al., 2010).

Mutation-Calling: Structural Variation

Structural variants were called from discordantly mapping paired-end reads from short insert data by using MAQ alignments as previously described (Campbell et al., 2008; Stephens et al., 2009). In order to improve sensitivity of detection, additional candidate structural variants were sought from within the proximity of copy number changes in the following way. All nontelomeric and noncentromeric segmentation breakpoints were obtained from SNP6 data processed via ASCAT (Van Loo et al., 2010). Candidate rearrangements close to copy number segmentation breakpoints were considered if rearrangements for which both breakpoints correspond to copy number breakpoints were identified or if one copy number breakpoint could only match one rearrangement. Rearrangements closer to a copy number breakpoint were preferred over rearrangements further away. When both rearrangement breakpoints matched a copy number breakpoint, the sum of the distances between the rearrangement and the copy number breakpoints was below 400kb, and when for only one rearrangement breakpoint a corresponding copy number breakpoint was found, the distance between both breakpoints was below 20kb.

Expression Analysis

Gene expression data were derived from the Illumina Human HT12 Expression BeadChip array, run in duplicate, normalized and processed as previously described (Pleasance et al., 2010).

Validation of Substitutions and Insertions/Deletions

Validation of putative somatic substitutions was performed via Roche pyrosequencing in 20 tumor-normal pairs and capillary sequencing in 1 tumor-normal pair (PD3890). All coding substitution variants and a random assortment of noncoding variants were selected for validation to make up to ~400 PCR products per sample. In addition to the variants validated to determine specificity, validation was also targeted to several hundred substitutions involved in regions of hypermutation and dinucleotides (Table S1B). Primers were designed to generate 275–425 bp fragments suitable for Roche 454 pyrosequencing. The specificity of the calling of substitution variants from the Illumina sequence reads, was determined from the proportion of calls confirmed as somatic when sequenced on this orthogonal platform (Table S1A).

For pyrosequencing data, an average coverage of ~657X was achieved for each validated variant. A minimum of 25 reads of mapping quality of at least 20 and base quality of 25 and above were required to report each variant. To be considered as somatic:

variants were required to be present in at least 5% of the reads in the tumor and not in the normal, or
if present at a low mutation burden of < 5%, required chi-square testing to assist in confirmation of somatic status.

This imposition of relatively strict criteria could potentially generate false negative calls (true somatic variants called as tumor wild-type) resulting in an underestimation of the specificity of substitution-calling.

Validation of putative indels was achieved by capillary re-sequencing of the tumor and normal pair. Capillary sequencing failed in ~20% variants. Two attempts at PCR validation was attempted for each genome. Indel variants were confirmed as somatic if they were present in the tumor traces and not present in the traces from the matched normal.

Validation of Somatic Rearrangement

Structural variants were confirmed by custom-designed PCR across the rearrangement breakpoint as previously described (Campbell et al., 2008) or by local reassembly.

For local reassembly, candidate rearrangements in regions of interest had been previously identified as rearrangements in close proximity to copy number changes. Discordantly mapping read pairs that were likely to span breakpoints, as well as a selection of nearby properly-paired reads, were grouped for each region of interest. Using the Velvet de novo assembler (Zerbino and Birney, 2008), reads were locally assembled within each of these regions to produce a contiguous consensus sequence of each region. Nearby properly-paired reads were added to increase coverage and to enlarge the resulting contigs. Heterozygous rearrangements, represented by reads from the rearranged derivative as well as the corresponding nonrearranged allele, were instantly recognizable from a particular pattern of five vertices in the de Bruijn graph component of Velvet. Exact coordinates and features of junction sequence (e.g., microhomology or nontemplated sequence) were derived from this. Assembly of homozygous rearrangements resulted in a single contig corresponding to an isolated vertex in the de Bruijn graph. The exact breakpoints were identified by aligning to the reference genome as though they were split reads.

Nonnegative Matrix Factorization

Nonnegative matrix factorization (NMF) is a mathematical approach that factorizes or decomposes a complex multidimensional data set in order to identify defining underlying signatures that make up the pooled data set.

In brief, a given matrix A of size $N \times M$ can be factorized into two nonnegative matrices, W and H (Lee and Seung, 1999). Matrix W has a size $N \times k$, whereas matrix H has a size $k \times M$, where k is the desired rank. In most cases, the two nonnegative matrices are insufficient to fully decompose the original data A and a residual matrix U, which can be used to evaluate the approximate reconstruction error, is required. The NMF equation could be written as

$$A = W * H + U,$$

where W and H are selected to optimally factorize or decompose the original matrix A. Usually, such factorization is achieved by finding the solution for

$$\min_{W \in \mathbb{R}_+^{N \times k} \; H \in \mathbb{R}_+^{k \times M}} \frac{1}{2} \|A - W * H\|_F^2$$

In this study, we consider matrix A to be the complex, pooled, multidimensional data set that is made up of 96 features (N) comprising mutation counts of each mutation type (C > A, C > G, C > T, T > A, T > C, T > G) at each 5′ and 3′ base context from 21 (M) breast cancer cases. Thus, matrix A has a size of 96×21. We decompose this data set into two matrices - W with size $96 \times k$ and H with size $k \times 21$ where k is the number of signatures that we are trying to model and identify. We perform NMF and use a model selection approach for $k = 2 \cdots 20$. An optimal decomposition and value of k was chosen based on the cophenetic correlation coefficient (a measure of how faithfully clustering approaches preserve pairwise distances and therefore dendrogram structures) and the average reconstruction error (Brunet et al., 2004).

NMF was performed by using a modified version of the publicly-available implementation (Brunet et al., 2004) of the multiplicative updated algorithm (Lee and Seung, 1999) and was repeated 1,000 times for each value of k. The cophenetic correlation coefficient

indicated reproducibility and stability for k values between 2 and 6 (Figure S2A). The cophenetic correlation fell sharply for $k > 6$ (less than 0.95, Figure S2A) indicating a lack of robustness when a decomposition exceeded 6 signatures for this data set. Given a value of k, each sample was reconstructed and compared to the observed data (Figure S2B). Error in reconstruction for each value of k was plotted (Figure S2C), and a dramatic reduction in the slope of the reconstruction error revealed that the model stabilized at five mutational signatures. As such, we selected to decompose the pooled mutation data set into five stable mutational signatures. A typical comparison between the reconstructed and observed mutation profile is given in Figure S2B. The concordance indicated that five signatures were sufficient to describe the general behavior of mutation profiles of the 21 breast cancer samples.

In theory, whereas NMF is able to highlight true mutational signatures that underlie mutational profiles in cancers, it should be noted that it is also able to identify systematic sequencing artifacts as a mutational signature.

Monte Carlo Simulation of Double Substitutions

In order to assess the degree of enrichment and statistical significance of the occurrence of double-nucleotide substitutions, Monte Carlo simulations were performed for each cancer genome. The mutation prevalence of each mutation type (C > A, C > G, C > T, T > A, T > C, T > G) was obtained for each chromosome of each cancer genome. For each genome, 1000 simulations were then performed by generating mutations in silico, at the observed mutation rates. For each simulation, the total number of in silico double substitution was identified and this number was compared to the observed number of double substitutions in the cancer genome. None of the simulations yielded a greater number of double-nucleotide substitutions than were observed in the cancer genomes, hence $p < 0.001$ for the observed enrichment of double substitutions for each cancer genome.

Germline Status of Breast Cancer Samples

Verification of germline mutation status was sought in those breast cancers reported as being derived from germline *BRCA1* and *BRCA2* mutation carriers. In addition, CaVEMan, Pindel and rearrangement outputs were screened for potential previously unidentified germline *BRCA1* and *BRCA2* mutation, in all the breast cancers. Germline mutation data are provided in Table S1A.

Relationship of CpG Methylation Status and Somatic Substitutions

In order to assess the relationship between methylation status of CpG dinucleotides and somatic substitutions, the mutation rates of C > T transitions occurring within CpG islands and outside CpG islands (http://genome.ucsc.edu/) were determined. The samples were considered in aggregate apart from PD4120a that was excluded because of the nature of the excessive mutation burden contributed by this sample. The odds ratio of the rate of mutation outside to inside CpG islands was calculated.

SUPPLEMENTAL REFERENCES

Bignell, G.R., Greenman, C.D., Davies, H., Butler, A.P., Edkins, S., Andrews, J.M., Buck, G., Chen, L., Beare, D., Latimer, C., et al. (2010). Signatures of mutation and selection in the cancer genome. Nature *463*, 893–898.

Brunet, J.P., Tamayo, P., Golub, T.R., and Mesirov, J.P. (2004). Metagenes and molecular pattern discovery using matrix factorization. Proc. Natl. Acad. Sci. USA *101*, 4164–4169.

Campbell, P.J., Stephens, P.J., Pleasance, E.D., O'Meara, S., Li, H., Santarius, T., Stebbings, L.A., Leroy, C., Edkins, S., Hardy, C., et al. (2008). Identification of somatically acquired rearrangements in cancer using genome-wide massively parallel paired-end sequencing. Nat. Genet. *40*, 722–729.

Kozarewa, I., Ning, Z., Quail, M.A., Sanders, M.J., Berriman, M., and Turner, D.J. (2009). Amplification-free Illumina sequencing-library preparation facilitates improved mapping and assembly of (G+C)-biased genomes. Nat. Methods *6*, 291–295.

Lee, D.D., and Seung, H.S. (1999). Learning the parts of objects by non-negative matrix factorization. Nature *401*, 788–791.

Li, H., and Durbin, R. (2009). Fast and accurate short read alignment with Burrows-Wheeler transform. Bioinformatics *25*, 1754–1760.

Pleasance, E.D., Cheetham, R.K., Stephens, P.J., McBride, D.J., Humphray, S.J., Greenman, C.D., Varela, I., Lin, M.L., Ordóñez, G.R., Bignell, G.R., et al. (2010). A comprehensive catalogue of somatic mutations from a human cancer genome. Nature *463*, 191–196.

Stephens, P.J., McBride, D.J., Lin, M.L., Varela, I., Pleasance, E.D., Simpson, J.T., Stebbings, L.A., Leroy, C., Edkins, S., Mudie, L.J., et al. (2009). Complex landscapes of somatic rearrangement in human breast cancer genomes. Nature *462*, 1005–1010.

Van Loo, P., Nordgard, S.H., Lingjærde, O.C., Russnes, H.G., Rye, I.H., Sun, W., Weigman, V.J., Marynen, P., Zetterberg, A., Naume, B., et al. (2010). Allele-specific copy number analysis of tumors. Proc. Natl. Acad. Sci. USA *107*, 16910–16915.

Ye, K., Schulz, M.H., Long, Q., Apweiler, R., and Ning, Z. (2009). Pindel: a pattern growth approach to detect break points of large deletions and medium sized insertions from paired-end short reads. Bioinformatics *25*, 2865–2871.

Zerbino, D.R., and Birney, E. (2008). Velvet: algorithms for de novo short read assembly using de Bruijn graphs. Genome Res. *18*, 821–829.

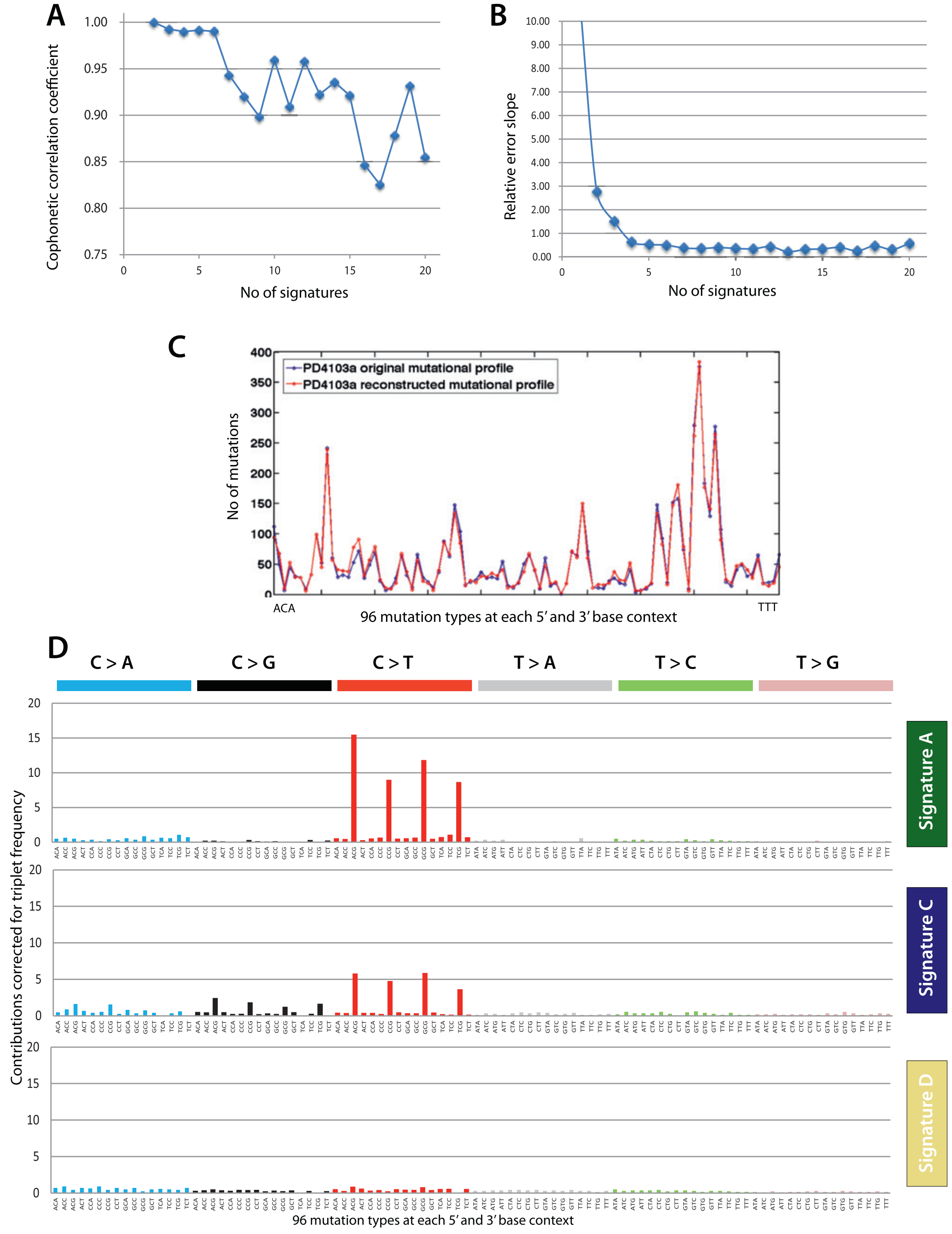

Figure S1. Selection of the Optimal Number of Signatures via the NMF Model Selection Framework, Related to Figure 2

(A) The x axis depicts the number of signatures, whereas the y axis shows the cophenetic coefficient. As an indicator of stable reproducibility, the cophenetic correlation coefficient is at its highest points at between 2 and 6 processes. Given that there are no further peaks after 6 for this data set, the number of signatures recognized by the NMF algorithm here is up to six.

(B) The error in reconstruction for each number of potential signatures, k, showed a marked reduction in the slope of the reconstruction error until k = 5, suggesting that the model was stable at five mutational signatures.

(C) A typical comparison between the reconstructed and original mutation profile demonstrating how well the extracted signatures and their exposures describe the original data for five signatures.

(D) Signatures A,C and D with contributions from each of the 96 trinucleotides corrected for the frequency of trinucleotides in the genome. This form of representation highlights the contrast between Signature A and C, as well as demonstrates the differences between Signatures C and D. Note the absence of C > T transitions at XpCpG in Signature D.

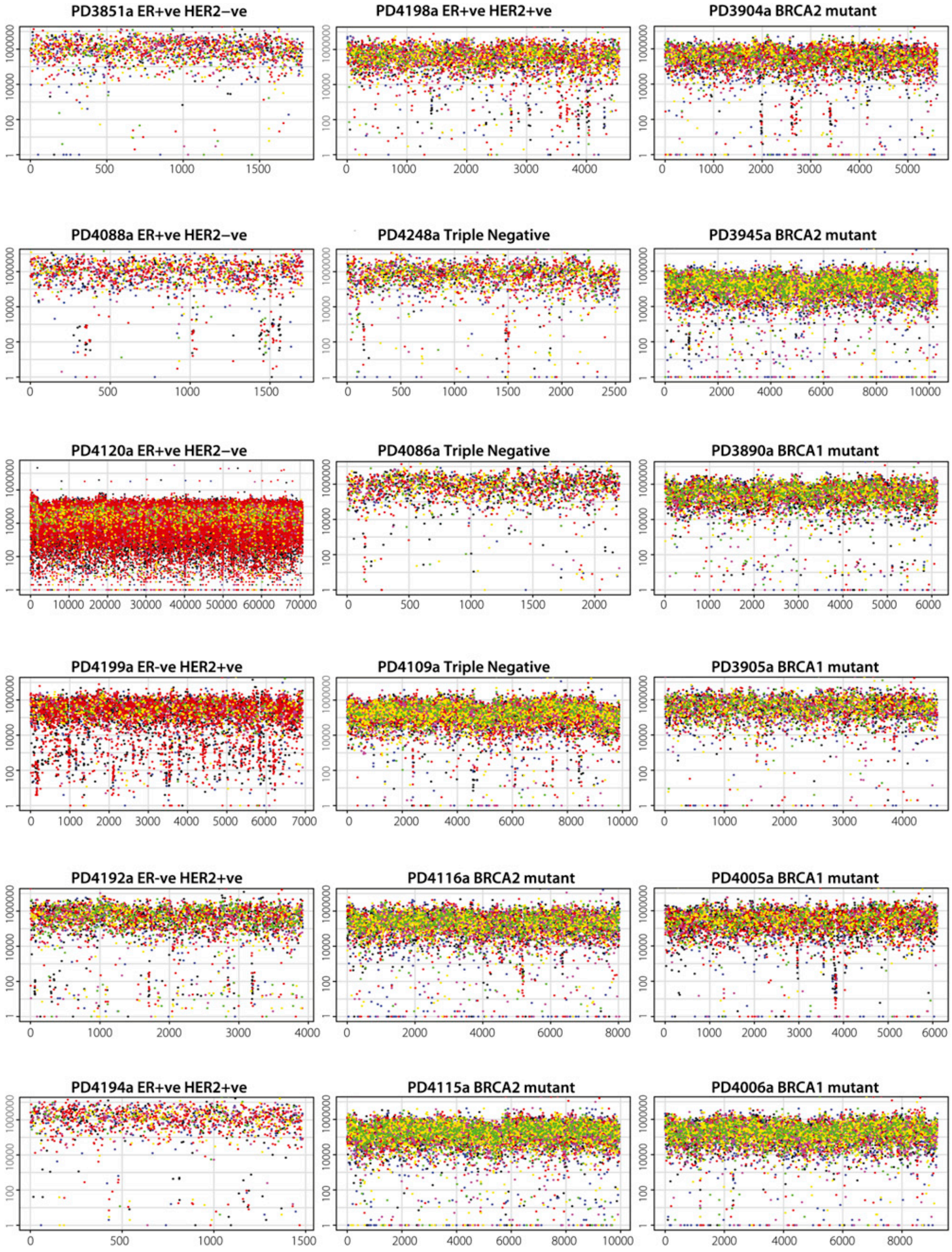

Figure S2. Rainfall Plots for 18 Genomes, Related to Figure 3

PD4115a, PD4116a, PD3904a, PD3945a, PD4005a and PD4006a show an excess of mutations of intermutation distance of 1bp, in-keeping with the observed excess of double substitutions in these genomes. Subtle regions of kataegis are present in many samples (PD4199a, PD4192a, PD4198a, PD4248a, PD4116a, PD3904a, PD4005a and PD4006a). Intermutation distance (bp) is presented on the vertical axis and mutation number is presented on the horizontal axis.

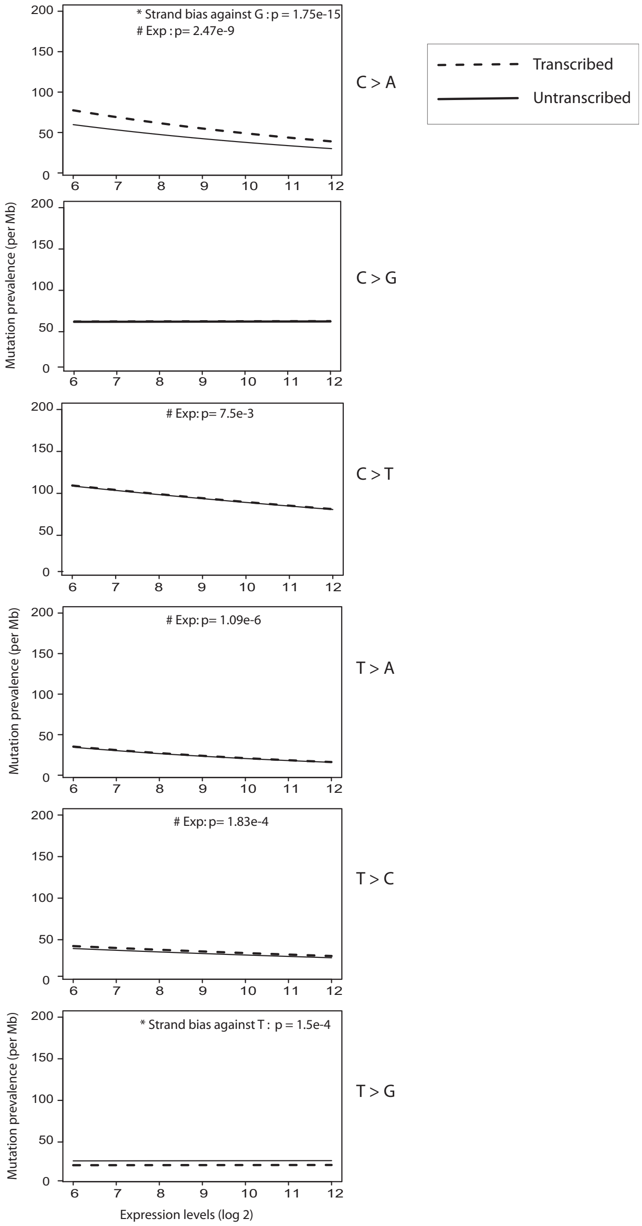

Figure S3. Relationship between Mutation Prevalence, Transcription and Gene Expression, Related to Figure 6

Overall effect of transcription and gene expression on mutation prevalence by mutation type. p values of significance are provided for each mutation-type if a strong effect was seen in either strand bias and/or relationship with expression. Mutation prevalence is expressed as the number of mutations per Mb from 0 to 2 per Mb on the vertical axis. Log 2 expression levels range from 6 to 12 on the horizontal axis. Lines are fitted curves to the data for A and B.
